# Microbial community structure of *Penaeus vannamei* post-larvae naturally infected with multiple viruses in hatchery tanks

**DOI:** 10.1371/journal.pone.0361576

**Published:** 2026-10-27

**Authors:** Betsy Andrade, Mery Ramírez, Guillermo Reyes

**Affiliations:** 1 Centro Nacional de Acuicultura e Investigaciones Marinas, CENAIM, Escuela Superior Politécnica del Litoral, ESPOL, Guayaquil, Ecuador; 2 Facultad de Ciencias del Mar, Universidad Península de Santa Elena, UPSE, Santa Elena, Ecuador; Universidad Nacional Autonoma de Mexico Instituto de Biotecnologia, MEXICO

## Abstract

**Background:**

This study offers an observational evaluation of how viral and bacterial infections co-occur with alterations in the microbiome and survival of *Penaeus vannamei* post-larvae, one of the most economically important species in global aquaculture.

**Methods:**

Eleven commercial larval production tanks were classified into three distinct infection scenarios using histology and PCR: *Baculovirus penaei* (BP) monoinfection, mixed infections (BP, white spot syndrome virus [WSSV], and acute hepatopancreatic necrosis disease [AHPND]-causing bacteria), and non-infected controls. High-molecular-weight DNA was subjected to shotgun metagenomic sequencing on the Illumina NovaSeq 6000 platform, followed by evaluation of microbial diversity, community structure, and infection-associated microbial signatures.

**Results:**

Although BP was the most abundant viral genus across infected groups, severe mortality was observed only under mixed-infection conditions, which were closely associated with AHPND-causing bacteria presence and microbiome disruption rather than with BP alone. Deep metagenomic sequencing revealed reduced, distinct microbiome structures in shrimp infected with BP and in those with mixed infections, compared to non-infected individuals. Non-infected post-larvae maintained complex and well-balanced bacterial communities, predominantly composed of *Donghicola* and *Muricauda*. Nudiviridae and Oscillospiraceae showed comparable relative abundances in BP-only and mixed-infection groups. In contrast, mixed infections were characterized by a relative increase in *Vibrio*, together with the PCR-detection of *PirAB* toxin genes, whereas WSSV remained detectable in the metagenomic dataset despite its comparatively low relative abundance, while the infection remained thermally suppressed and asymptomatic in the larvae. Functionally, infected larval microbiomes were enriched for transposases, retrotransposons, and virulence-factor genes. The near-complete BP-GR25 genome (119,245 bp) was assembled and annotated from larval metagenomes.

**Conclusion:**

These results highlight the value of high-throughput sequencing in the early detection of pathogens in aquaculture, where subtle microbial shifts during postlarval stages can be valuable indicators of health status for production.

## Introduction

Shrimp farming began in Asia during the 1970s and has expanded steadily to the present day. For some producer countries in South America, shrimp aquaculture accounts for a major share of the national economy. However, this growth has been accompanied by an increase in the incidence of infectious diseases affecting farmed populations [1–3]. *Baculovirus penaei* (BP) or *Penaeus vannamei* singly enveloped nuclear polyhedrosis virus (PvSNPV) has recently been reallocated to the Nudiviridae family [4]. This enteric agent predominantly targets the epithelial cells of the hepatopancreas and anterior midgut [5–7]. This pathogen can affect several penaeid shrimp species throughout all life stages. Early ontogenetic phases “larval and postlarval up to postlarva 9 (PL9)” show extreme vulnerability, with mortality typically observed within 2–5 days post-exposure [8,9].

Global shrimp production is also heavily pressured by white spot syndrome virus (WSSV) [10]. This pathogen is renowned for its rapid spread, elevated mortality rates, and ability to infect shrimp at all developmental stages—from larvae to adults [11,12], making it a primary driver of massive financial losses across the aquaculture sector worldwide [13]. Compounding these viral concerns is *Vibrio parahaemolyticus*, the etiological agent of acute hepatopancreatic necrosis disease (AHPND). The virulence of this bacterial pathogen stems from strains that harbor the binary toxins *PirA* and *PirB*, which induce severe structural degradation and sloughing of the hepatopancreatic tubule epithelium [14–18].

Pathogenicity and mortality rates in penaeid shrimp are often intensified during concurrent bacterial and viral infections. A prime example of this detrimental relationship involves the co-occurrence of *Vibrio parahaemolyticus* and white spot syndrome virus (WSSV). Additionally, it has been suggested that BP infections may be associated with an increased incidence of secondary bacterial infections [19–21]. These findings suggest that disease in shrimp may result from the combined action of multiple infectious agents rather than a single pathogen.

Consequently, the endogenous microbiome of penaeid shrimp constitutes a vital determinant of overall fitness. This microbial shield safeguards host health through its multifaceted contributions to nutrient assimilation, immune system modulation, and robust defense against opportunistic outbreaks [22–29]. However, pathogens may persist at low relative abundances in healthy individuals without causing disease [29,30], indicating that the onset of pathological states is more closely associated with imbalances in the microbial community and environmental factors than with the mere presence of potentially pathogenic microorganisms [31].

By shifting away from classical etiological models centered on a solitary causative agent, this viewpoint embraces the principles of polymicrobial pathogenesis. Under this lens, pathological states are viewed as systemic outcomes driven by the intricate interplay between multiple co-existing microorganisms and their surrounding ecological context [31–33]. In this sense, the pathobiome approach provides an integrative framework for analyzing these interactions at the community level, considering both microbial composition and functional potential, including virulence factors, to determine disease outcomes [34].

Despite these advances, the roles of specific viral agents within the shrimp microbiome—particularly BP—and their relationships with microbial diversity and co-existing pathogens remain poorly understood. In particular, the relative abundance of BP and its association with other pathogens, such as white spot syndrome virus (WSSV) and AHPND-causing bacteria, have not been systematically investigated under real production conditions.

This study aimed to characterize the microbiome structure of *P. vannamei* under single- and mixed-infection conditions (BP–WSSV and AHPND-causing bacteria) in commercial hatchery tanks using high-throughput DNA sequencing (HTS) to identify microbial signatures potentially associated with these infection states.

## Materials and methods

### Sample collection, diagnosis, and classification of infection status

To capture a representative snapshot of the system, and considering the individual tank as the experimental and statistical unit, *Penaeus vannamei* post-larvae (PL10 stage) were sampled in parallel from 11 commercial-scale production tanks, each possessing a 35-ton capacity. The collection was carried out at a commercial hatchery facility in South America with a well-documented record of persistent viral outbreaks. All tanks were managed under the same feeding protocol throughout the production cycle. For each tank, the post-larvae analyzed represented a pooled biological sample. Each sample was divided into two subsamples for distinct analyses: (1) approximately 1 g of randomly collected larvae (representing approximately 250 individuals) was fixed in Davidson’s solution and processed for histopathological examination, and (2) approximately 1 g of larvae was macerated and preserved in DNA/RNA Shield solution at −20 °C for subsequent pathogen PCR diagnostics and HTS-based microbiome analysis.

Environmental and production variables across the 11 tanks (detailed in S1 Table) were statistically compared among groups to assess potential confounding effects. Data were analyzed using analysis of variance (ANOVA) after verifying normality (Shapiro-Wilk test) and homoscedasticity (Levene’s test). Differences among group means were identified using Tukey’s honest significant difference (HSD) post-hoc test.

Tissue sections from 30 larvae were stained with Mayer-Bennet H&E for histopathological examination. Among the 11 tanks analyzed, three distinct histopathological patterns were identified in *P. vannamei* larvae. (1) A group of tanks contained larvae showing tetrahedral occlusion bodies within hepatopancreatic cells and the intestinal lumen, consistent with BP infection. (2) Another group exhibited a mixed infection pattern, in which the larval population showed tetrahedral occlusions typical of BP infection and detached and necrotic hepatopancreatic cells characteristic of acute hepatopancreatic necrosis disease (AHPND). In contrast, (3) larvae from the remaining group of tanks did not display any histopathological lesions.

Corresponding tank samples were then selected for PCR diagnostic. The preserved macerated samples were thawed, and DNA was extracted using the phenol–chloroform method. Pathogen screening was executed via nested PCR assays utilizing specific diagnostic primers: the BP set [35], IK1/2 - IK3/4 targeting WSSV [13], SWP 1F/1R – SWP 2F/2R for EHP [36], NHPF2/R2 for NHP [37], and IHHNV309F/R for IHHNV detection [38]. Furthermore, bacterial strains responsible for AHPND were identified using the AP4 primer set [39]. Positive and negative controls were included in all PCR diagnostic assays.

Based on PCR and histology results, the samples were classified as follows: four tanks were infected with BP; four tanks were co-infected with mixed infections (i.e., BP, WSSV, and AHPND-causing bacteria); and the remaining three tanks were negative for the analyzed pathogens. To facilitate microbiome analysis, frozen post-larvae representing both the non-infected and infected groups were thawed and thoroughly homogenized under liquid nitrogen (−196 °C) into a fine powder.

### DNA extraction and microbiome sequencing

Extraction of total genomic DNA was performed using the Quick-DNA HMW MagBead Kit (Zymo Research, USA) according to the manufacturer’s protocol. To evaluate the concentration and structural integrity of the recovered DNA, samples were analyzed using a Quantus fluorometer (Promega, USA) and verified via agarose gel electrophoresis. The extracted DNA showed an A260/230 ratio of ~2.0 and a concentration of ~30 ng/µL, with electrophoresis revealing high-molecular-weight DNA bands of ~1 kb. High-throughput sequencing was performed on an Illumina NovaSeq 6000 platform to process the shotgun metagenomic libraries using a PE150 strategy. These raw sequencing data are publicly available in the Short Read Archive (SRA) repository at NCBI under the accession number PRJNA1452228.

### Metagenome assembly and annotation

Prior to assembly, host-derived reads were systematically depleted using Kneaddata (v0.12.0; https://github.com/biobakery/kneaddata) with a custom database containing the *P. vannamei* genome (NCBI RefSeq Assembly ID: GCA_042767895.1) via Bowtie2 (--very-sensitive mode). Per-sample read statistics across processing steps are provided in S2 Table. Retained host-depleted metagenomic reads were processed using the SqueezeMeta pipeline (v1.7.2) [40] hosted on a local server at CENAIM. The analysis was conducted in co-assembly mode, employing Megahit (v1.2.9) for *de novo* assembly [41] with default parameters and a filtering cutoff for contigs shorter than 500 bp. To establish the taxonomic identity of the resulting contigs, the integrated Lowest Common Ancestor (LCA) algorithm was executed under standard SqueezeMeta settings. Bowtie2 (v2.5.4) was used to back-map sequencing reads from each sample onto the generated contigs, facilitating precise estimation of contig-specific coverage and abundance. The raw number of reads mapped to each contig was extracted using BEDtools. To isolate targeted viral sequences, deepVirFinder [42] was applied to filter contigs with a minimum length of 3,000 bases. The resulting data suites (taxonomy, abundance tables, and metadata) were first structured as a phyloseq instance and then parsed into a microtable object through the microeco package (v2.3.0) [43]. Finally, a Cumulative Sum Scaling (CSS) normalization was applied to the mapped read count matrix to prepare it for subsequent comparative analyses.

### Microbiome diversity and differential abundance

Alpha diversity metrics, including the abundance-based coverage estimator (ACE) and the Shannon index, were used to assess microbial diversity across health statuses and statistically compared with the Kruskal-Wallis test. Taxonomic profiling was conducted by computing the relative abundance of microbial communities within each group across multiple hierarchical ranks, specifically at the kingdom, phylum, family, and genus levels. Furthermore, beta-diversity variations among the cohorts were evaluated using the Bray-Curtis dissimilarity index and projected via principal coordinate analysis (PCoA) to identify underlying structural patterns. Differences in microbiome composition between health statuses were evaluated using PERMANOVA. To identify distinctive microbial signatures, genus-level abundance differences among groups were evaluated using the Kruskal–Wallis test, with *p*-values adjusted using the Benjamini–Hochberg false discovery rate (FDR) correction.

### Microbiome function and associated virulence factors

Gene prediction was performed on the assembled contigs using Prodigal (v2.6.3) for protein-coding genes, and Barrnap (v0.9) for rRNA gene detection. The resulting open reading frames (ORFs) were functionally annotated. Homology research was conducted against the GenBank nr database using DIAMOND (v2.1.14) [44] for taxonomic assignment, and against the COG and Pfam databases using HMMER (v3.4) [45]. Functional count tables were generated using the SQMtools R package based on the annotated ORFs obtained from SqueezeMeta. Functional abundances were represented as mapped metagenomic read counts, and the resulting functional count matrix was normalized using Cumulative Sum Scaling (CSS) prior to comparative analyses. Functional dissimilarities among samples were evaluated using PERMANOVA, and the Kruskal-Wallis test was applied to assess differences in abundance between groups. In parallel, the annotated ORFs from all samples were filtered to identify virulence factor genes (VFGs).

### *Baculovirus penaei* genome assembly

Through binning with DAS Tool (v1.1.7), the genome of *Baculovirus penaei* BP-GR25 was recovered from larval microbiome samples. Genome quality was evaluated with CheckV (v1.1.1) [46], while annotation and identification were performed using the VAPiD pipeline (v1.2) [47], complemented by a manual search for coding regions in BLAST-NCBI [48]. To evaluate sequence similarity at the whole-genome level, an Average Nucleotide Identity (ANI) analysis was executed via fastANI (v1.34). The genomic architecture was visualized as a circular map using the Genovi pipeline (v0.2.16) [49]. Furthermore, evolutionary relationships were investigated through a genome-scale phylogenomic analysis executed within the PhyloSuite platform (v2.0) [50]. Public access to the near-complete BP-GR25 genome is provided via the GenBank repository, where it was registered under the specific accession number PZ441674. To independently validate the BP-GR25 genome across samples and assess potential strain-level variation, host-depleted paired-end metagenomic reads from each sample were independently mapped against the 119,245-bp BP-GR25 reference genome using Bowtie2 (v2.5.4) with the --very-sensitive alignment preset. The resulting alignments were converted to BAM format, sorted, and indexed using SAMtools (v1.24). Per-sample mapping statistics were calculated, including the number of mapped reads, mapping rate, genome breadth (percentage of reference positions covered by at least one read), mean and sequencing depth. Sequence variation relative to BP-GR25 was assessed using BCFtools (v1.24) through pileup generation and variant calling. High-quality SNPs were retained using minimum thresholds of read depth ≥10 and variant quality (QUAL) ≥30, and SNP density was calculated relative to the BP-GR25 genome length.

## Results

### Sample collection, diagnosis, and classification of infection status

Histopathological examination of *P. vannamei* post-larvae revealed three distinct tissue response patterns across the 11 production tanks. Four tanks contained larvae with tetrahedral occlusion bodies in hepatopancreatic cells and in the intestinal lumen (Fig 1), characteristic of BP infection.

**Fig 1:**
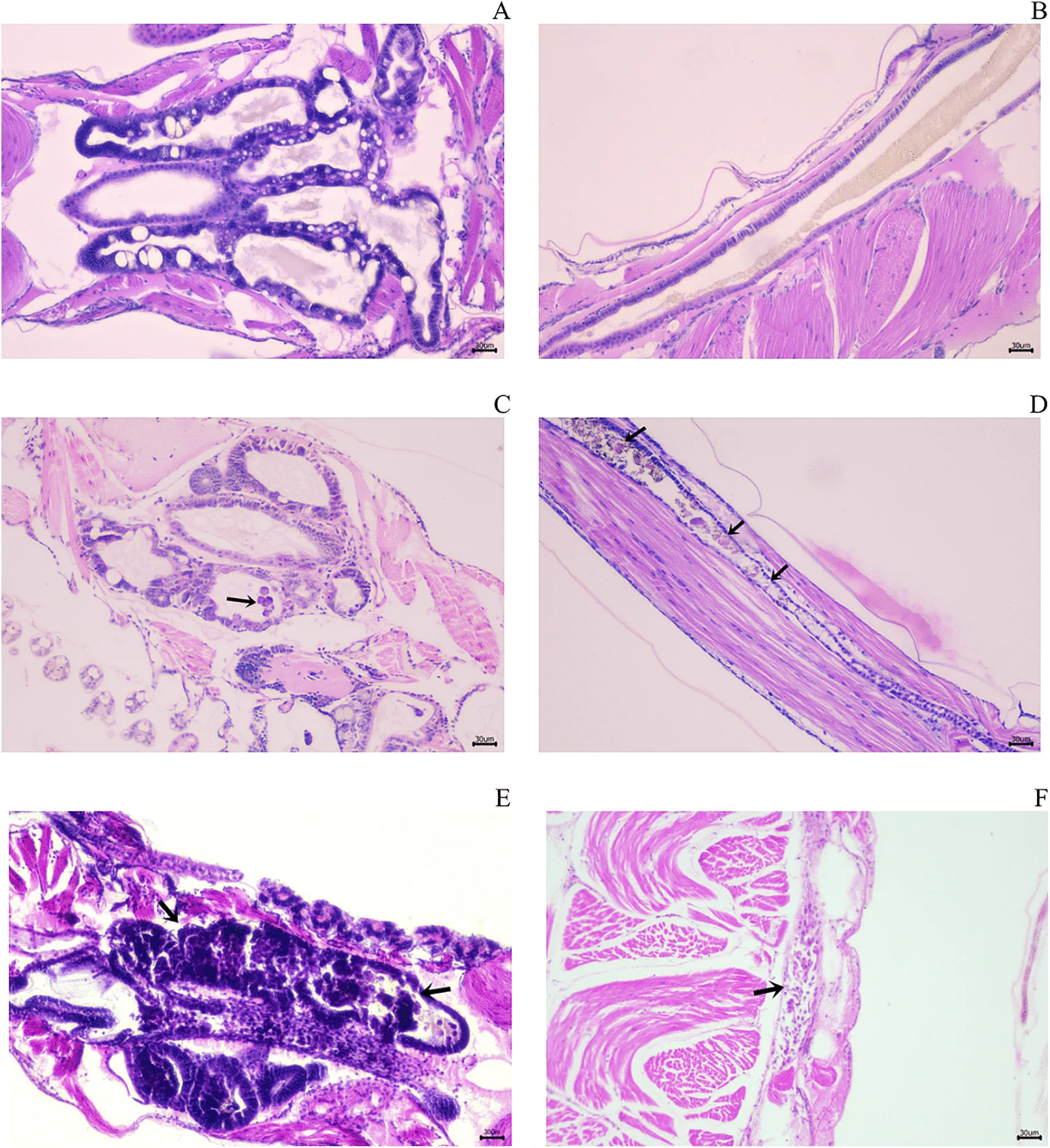
Histological section of *Penaeus vannamei* post-larvae (PL10) stained with Mayer-Bennet hematoxylin and eosin. (A) Hepatopancreatic tubules under normal conditions, 10 × . (B) Midgut epithelium under normal conditions, 10 × . (C) Hepatopancreatic tubules showing tetrahedral occlusion bodies characteristic of *Baculovirus penaei* (BP), black arrow, 10 × . (D) Midgut epithelium containing tetrahedral occlusion bodies characteristic of BP, black arrow, 10 × . (E) Hepatopancreatic epithelial cell detachment characteristic of the terminal phase of AHPND. (F) Intestinal epithelial cell detachment characteristic of the terminal phase of AHPND.

In this group, tetrahedral occlusion bodies were observed at a prevalence of 33.33 ± 5.77%. A second group of tanks (n = 4) showed mixed lesions, with some larvae exhibiting BP-like occlusions and others showing detached and necrotic hepatopancreatic epithelial cells, indicative of acute hepatopancreatic necrosis disease (AHPND), hereafter referred to as “Mixed infection group”. In this group, the prevalence of BP was 30.0 ± 12.86%, and that of AHPND-related lesions was 55.0 ± 7.07% (S3 Table).

Lesions associated with WSSV were not observed by histopathology analysis but were detected by PCR (Table 1 and S4 Table). The remaining three tanks displayed no apparent histopathological alterations and had samples with no detectable pathogens based on histology and PCR, hereafter referred to as the “non-infected group”. In the post-larvae samples analyzed, no other viruses or bacteria were detected beyond the pathogens identified in each infected group. At the PL11 harvest, survival differed significantly among groups, with mixed-infections tanks showing the lowest survival (27.5 ± 1.1%), followed by BP-infected tanks (63.3 ± 2.2%), and non-infected tanks (70.8 ± 0.9%). Tukey’s HSD post-hoc test confirmed significant differences for all pairwise comparisons (*p* < 0.001). In contrast, no significant differences were detected among groups for dissolved oxygen, temperature, salinity, and pH (ANOVA, *p* > 0.05), indicating that the monitored environmental parameters did not differ significantly among infection groups.

**Table 1:** Comparison of histological findings and PCR diagnostic results across 11 sampled tanks. PCR results are expressed as the number of PCR-positive tanks over the total number of tanks tested within each group (n/N). WSSV, white spot syndrome virus; AHPND, acute hepatopancreatic necrosis disease; BP, *Baculovirus penaei.*

Group	*n*	Histological analysis	PCR Results (WSSV / AHPND-causing bacteria / BP)	Key Observations	Survival at harvest (PL11)
**BP-infected**	4	*Baculovirus* (BP)	WSSV (0/4) / AHPND (0/4) / BP (0/4)	Limited BP detection by PCR	63.3 ± 2.2%
**Mixed infection**	4	BP + AHPND	WSSV (4/4) / AHPND (4/4) / BP (1/4)	Full detection of AHPND/WSSV; limited BP amplification.	27.5 ± 1.1%
**Non-infected**	3	No lesions	WSSV (0/3) / AHPND (0/3) / BP (0/3)	Total agreement between methods.	70.8 ± 0.9%
**Total**	**11**				

### Metagenome assembly and annotation

Metagenomic sequencing yielded a total of 732 million clean reads across the 11 *P. vannamei* larval samples, averaging 67 million reads per sample. Following co-assembly, 2,9 million contigs were generated (longest contig: 523,334 bp). Gene prediction identified 3,433,465 protein-coding ORFs using Prodigal. In addition to protein-coding genes, RNA gene prediction identified 11,504 tRNA/tmRNA sequences with Aragorn and 933 rRNA genes with Barrnap. Regarding functional databases, 584,010 ORFs (17.0% of the predicted ORFs) received KEGG functional assignments, representing 11,793 KEGG entries, whereas 849,354 ORFs (24.7%) were assigned to COG categories, encompassing 35,393 COG entries. Genome-resolved reconstruction recovered 108 metagenome-assembled genomes (MAGs), of which 15 met high-quality criteria (>90% completeness and <10% contamination) and 25 met good-quality criteria (>75% completeness and <10% contamination).

### Microbiome diversity and differential abundance

Fig 2 displays the taxonomic composition of the studied metagenomes. To focus solely on the bacterial and viral communities, host-derived contigs were removed from the analysis. Fig 2A shows that the proportions of bacteria and viruses differ significantly across health statuses. In addition to the dominant bacterial phyla like Pseudomonadota, Bacillota, and Bacteroidota, samples infected with BP, as well as those with mixed infections (i.e., BP, WSSV, and AHPND-causing bacteria), showed a higher relative abundance of viral reads, primarily assigned to the class Naldaviricetes and the family Nimaviridae (Fig 2B).

**Fig 2:**
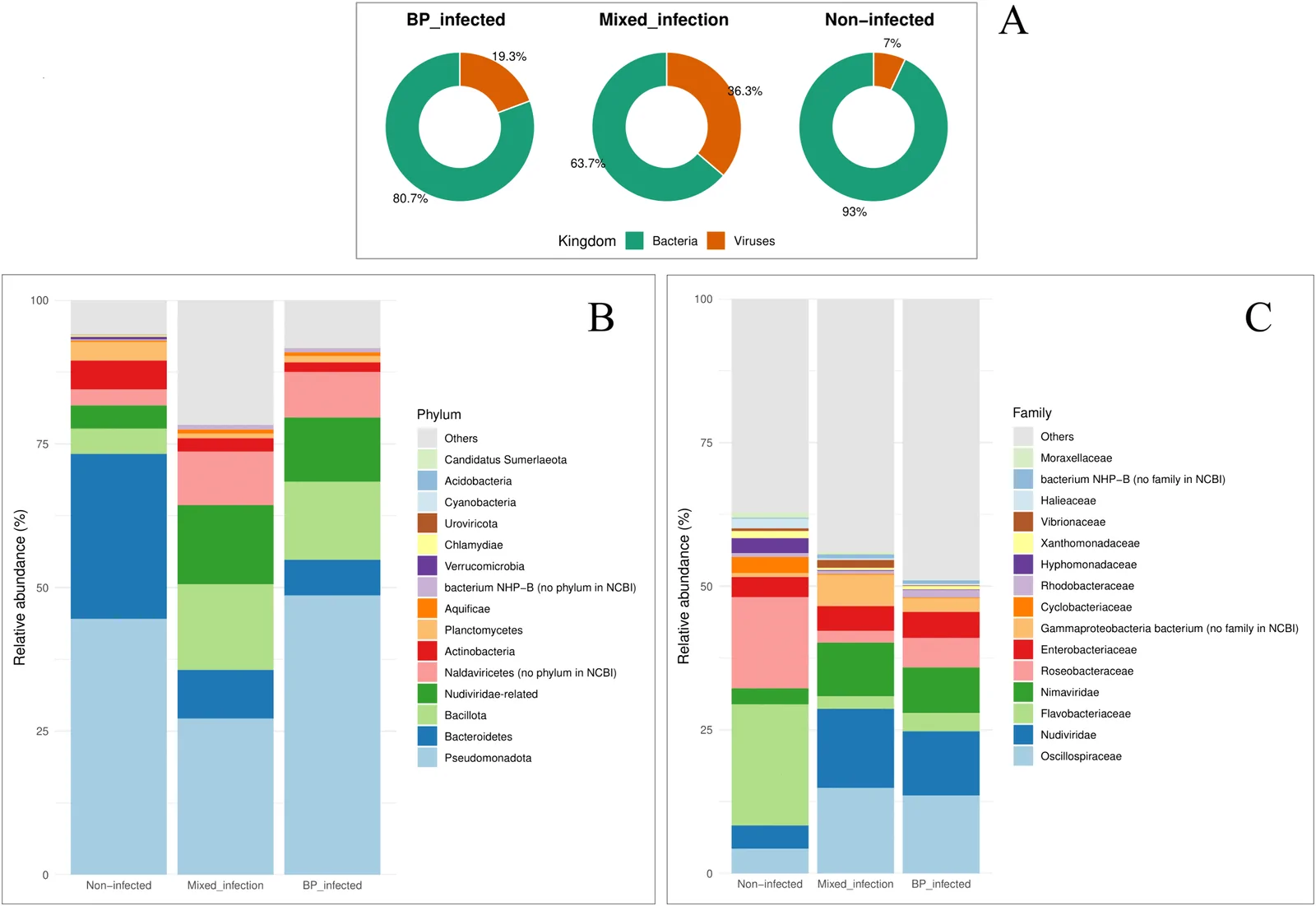
Taxonomic composition of the microbiome in *Penaeus vannamei* post-larvae (PL10) under different health status (non-infected, mixed infection: *Baculovirus penaei*, WSSV, and AHPND-causing bacteria, and infected solely by BP). (A) Relative proportion of reads assigned to bacteria and viruses in each health condition. (B) Relative abundance of the main bacterial and viral phyla, and (C) Relative abundance at the family level.

Unlike infected groups, the non-infected group displayed a microbiome dominated nearly exclusively by bacteria, with very little viral representation and a unique taxonomic structure. Additionally, NHP-associated bacteria were detected at low abundance in both infected groups, accounting for 0.62% and 0.67%, respectively.

Both infected groups clustered together phylogenetically, suggesting greater similarity in microbial community structure (Fig 3A). At the genus level, *Donghicola* and *Muricauda* (bins/MAGs with >90% completeness) were predominant in the non-infected group, with relative abundances of 11.03% and 10.19%, respectively (Fig 3B). In contrast, a member of the Oscillospiraceae family was the most abundant in the infected groups, reaching approximately 14% in each status.

**Fig 3:**
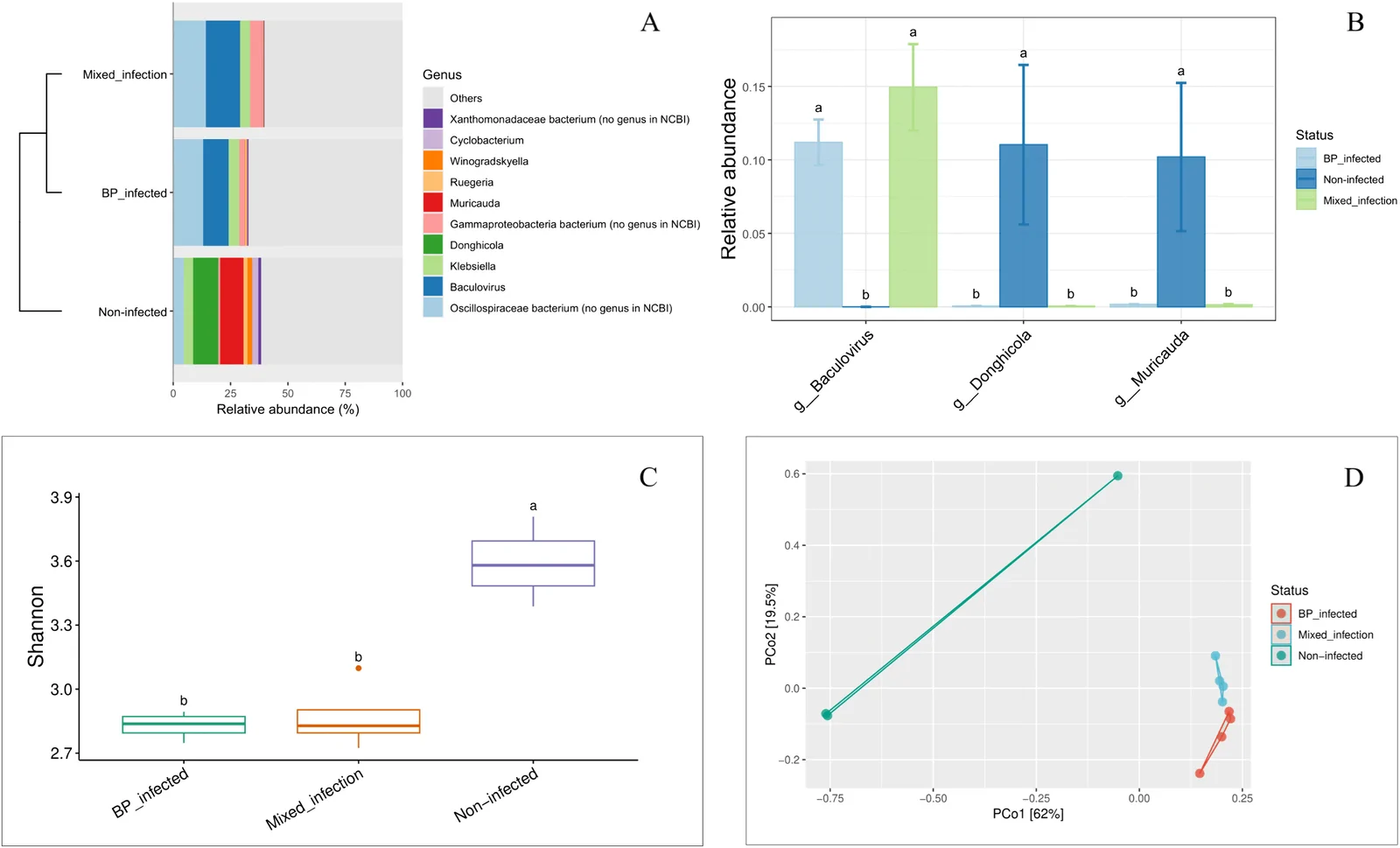
Microbial community composition and diversity in *Penaeus vannamei* post-larvae (PL10) under different health statuses. (A) Relative abundance of dominant bacterial genera in non-infected, mixed infection, and BP-infected groups, based on high-throughput sequencing. Genera accounting for > 1% relative abundance are shown. (B) Differential abundance of key genera (*Baculovirus*, *Donghicola*, and *Muricauda*) among the three health conditions. Different letters indicate statistically significant differences (Kruskal-Wallis test, *p* < 0.05). (C) Comparison of alpha diversity (Shannon index) across groups, showing a significant reduction in microbial diversity in infected conditions. Letters denote significant pairwise differences. (D) Principal Coordinate Analysis (PCoA, Bray–Curtis distance) illustrating clear segregation of microbial communities according to health status, with non-infected samples clustering apart from mixed and BP-infected groups.Overall, *Vibrio* represented a relatively small fraction of the shrimp microbiome (1.33% to 0.12%), although its relative abundance and community composition varied among health statuses. A relative increase in *Vibrio* was observed in the mixed-infection group, reaching a 3.2-fold higher abundance than in the non-infected group. In contrast, *Vibrio* abundance was lower in the BP-only group than in the non-infected group. Despite this lower abundance, *Vibrio* community composition differed significantly between BP-only and non-infected groups (PERMANOVA, *p* = 0.045).

Among the detected viral taxa, *Baculovirus* was the most abundant in the infected groups (Kruskal-Wallis test, *p* < 0.05), showing a 3.3-fold higher abundance than in the non-infected group. BLAST analysis of the *Whispovirus* bin against the NCBI database revealed high similarity to White Spot Syndrome Virus (WSSV). This virus was observed in both groups, representing approximately 1% of the total microbiome abundance.

According to the ACE index (Kruskal-Wallis test, *p* < 0.05), bacterial and viral richness were significantly lower in the infected groups than in the non-infected groups, which exhibited stable and diverse microbial communities. Moreover, both the mixed-infection and BP-infected groups showed significantly lower diversity, as indicated by the Shannon index (Kruskal-Wallis test, *p* < 0.05; Fig 3C). Consistently, Bray–Curtis dissimilarity-based β-diversity analysis revealed a clear separation between non-infected and infected groups (PERMANOVA, *p* = 0.001, Fig 3D).

### Microbiome function and associated virulence factors

Infected samples were significantly enriched in retrotransposon proteins, transposases, inactivated derivatives, and reverse transcriptases, among other COGs, albeit at lower but significant abundance (Fig 4A).

**Fig 4:**
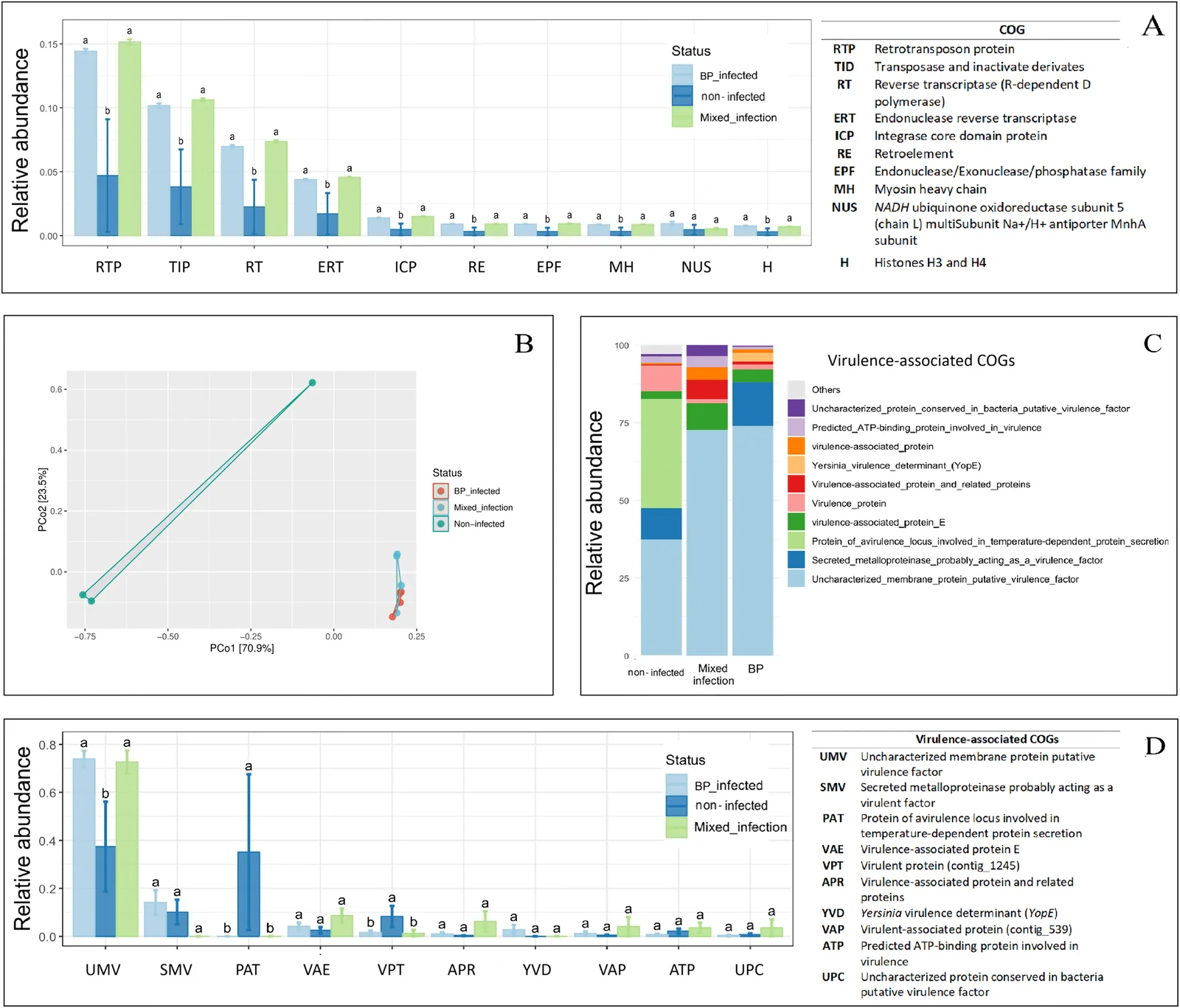
Functional and virulence-associated COG profiles in the microbiome of *Penaeus vannamei* post-larvae (PL10) under different health statuses. (A) Relative abundance of the most represented COG functional categories in each health status. (B) Principal Coordinate Analysis (PCoA) based on Bray–Curtis dissimilarity showing a clear separation between groups according to functional profiles (PERMANOVA, *p* < 0.001). (C) Distribution of virulence-associated COGs. (D) Differential abundance of individual virulence-associated COGs between groups.

These functions were associated predominantly with microbial/mobile genetic element-related sequences rather than residual shrimp genomic DNA, which had been removed during the host-depletion step prior to metagenomic assembly. Because these COGs are not specific to viral genomes, they were not directly attributed to baculoviruses or nudiviruses. The overall functional composition (COG profiles) differed significantly between infected and non-infected groups, as shown by PCoA based on Bray–Curtis dissimilarity (PERMANOVA, *p* < 0.001; Fig 4B). The functional profiling based on KEGG Orthology (KO) revealed a clear difference in the distribution of identified functions between non-infected and infected groups. In non-infected samples, multiple functional categories were consistently detected, including signaling and cellular processes (e.g., bacterial motility proteins, two-component systems, transporters, exosomes) and genetic information processing functions such as chromosome-associated proteins, transcription factors, DNA repair and recombination proteins, membrane trafficking, and mitochondrial biogenesis. Each of these categories contributed to a modest but detectable relative abundance, collectively representing around 10–15% of the total functional potential. In contrast, the mixed-infection and BP-infected groups showed a significant reduction (Kruskal-Wallis test, *p* < 0.001) in the number and relative abundance of identified KO functions.

When focusing on virulence-associated COGs, we observed a predominance of uncharacterized membrane proteins with putative virulence functions (Fig 4C), particularly in the infected samples (*p* < 0.001). Conversely, avirulence locus proteins involved in temperature-dependent protein secretion were significantly more abundant in the non-infected group (*p* < 0.05, Fig 4D). Analysis of protein domain composition using Pfam revealed that bacterial virulence factors such as hemolysin and SrfB were dominant in the mixed infection samples (*p* < 0.001).

### *Baculovirus penaei* genome

The *Baculovirus penaei* BP-GR25 genome is 119,245 bp in length, with a GC content of 33.3% and an average read coverage of 42x. Homology-based gene prediction identified a total of 122 ORFs, of which 91 were CDSs (Fig 5A, S5 Table). CheckV analysis validated the assembly as a high-quality viral genome with an estimated completeness of 96.13%. Independent mapping of host-depleted reads from each tank against BP-GR25 further supported the integrity of the recovered genome. Two samples from the mixed-infection group independently covered 100% of the 119,245-bp BP-GR25 genome, with mean sequencing depths ranging from 55.2× to 81.6 × , while a third mixed-infection sample showed 99.99% genome breadth at a mean depth of 32.1× (S6 Table). No high-quality SNPs were detected in samples with sufficient genome coverage for reliable variant assessment. The near-complete to complete genome-wide coverage obtained independently from these samples, together with the absence of detectable sequence variation, supports BP-GR25 as a coherent and highly conserved viral genome rather than a chimeric sequence resulting from the co-assembly. The genome possesses high COG abundance in lipid transport and metabolism, signal transduction, intracellular trafficking, secretion, and vesicular transport, replication, recombination, and repair, among other unknown functions (Fig 5B). The genome showed average nucleotide identity (ANI) values of 99.45% with *Penaeus vannamei nudivirus* (GenBank accession OM066077.1) and was clustered within *Penaeus monodon nudivirus* (Fig 5C).

**Fig 5:**
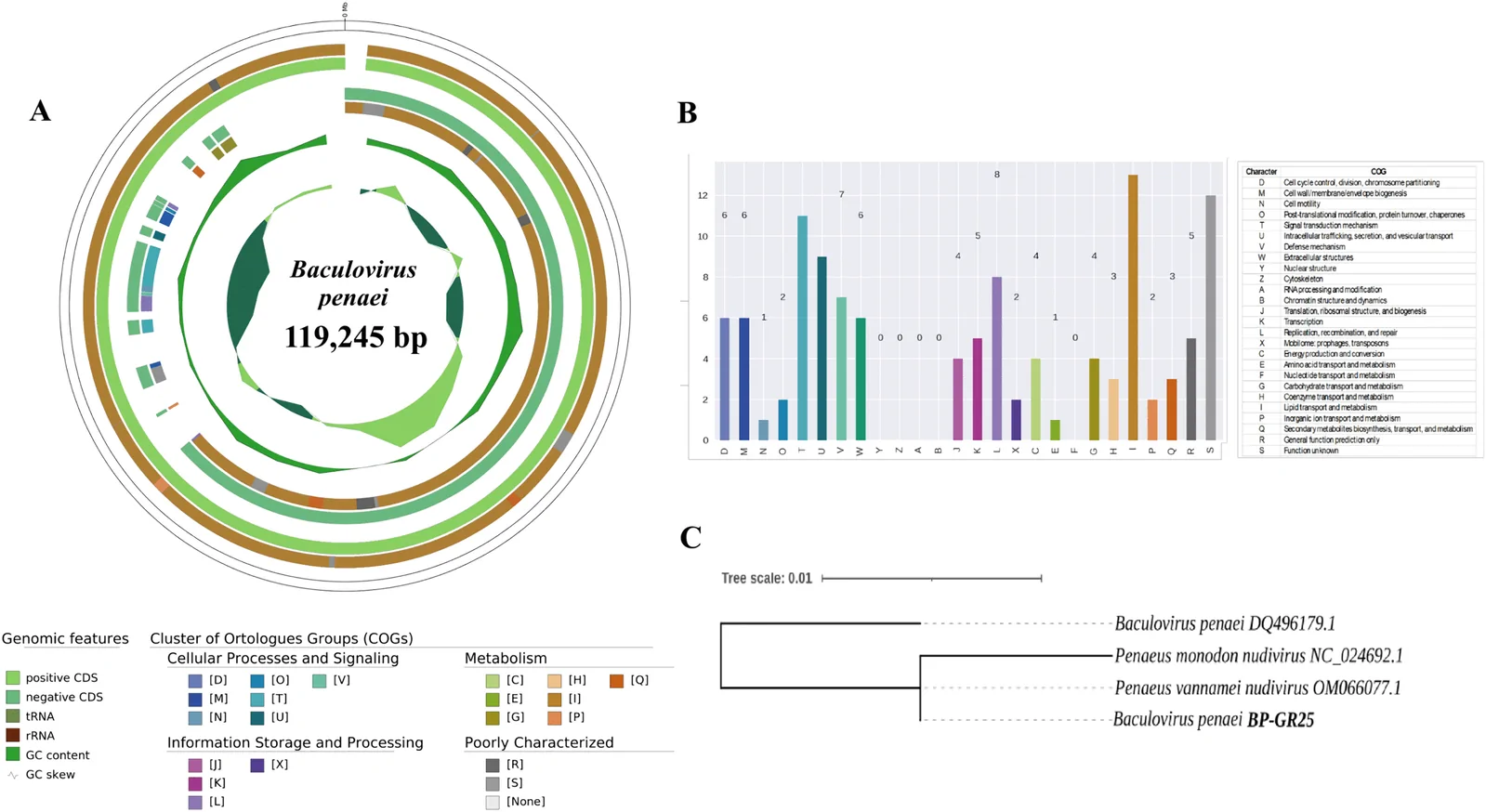
(A) Genome map of *Baculovirus penaei* (BP) strain BP-GR25. Circles from inside to outside: GC skew, GC content, rRNA, tRNA, negative CDS, and positive CDS. The functional categories of genes are annotated based on the COG database. The accompanying table provides a detailed explanation of the color codes, and (B) Distribution of clusters of orthologous groups of proteins (COGs). Each COG grouping of the coding sequences (CDSs) was assigned a category from A to Z. (C) Phylogenetic tree of nudivirus genomes, including the most closely related nudivirus strains.

## Discussion

Studies conducted on the shrimp *Penaeus vannamei* infected with *Baculovirus penaei*, or PvSNPV, under experimental conditions have shown that the stages from mysis 2 to post-larva 25 are vulnerable to infection [5,19,51]. The most vulnerable stage has been identified as post-larva 9, which usually shows clinical symptoms two to three days after viral exposure [6–8].

The consistent detection of BP in mixed infection with bacterial pathogens has been reported previously, suggesting a possible synergistic interaction in which bacterial toxins or local inflammatory processes may favor viral replication or the formation of inclusion bodies [19]. In our study, we observed two infection patterns across tanks: BP infection alone and mixed-infection conditions in which BP occurred together with WSSV and/or AHPND-causing bacteria. These infection patterns were supported by histological, molecular, and metagenomic evidence at the tank level. Mixed-infection tanks showed a relative increase in *Vibrio* compared with both BP-infected and non-infected groups. High mortality was restricted to mixed-infection tanks, indicating that BP alone was not linked to high mortality under these conditions. Although BP infection alone was associated with the lowest *Vibrio* abundance, *Vibrio* community composition differed significantly between BP-infected and non-infected groups, suggesting that BP may be associated with changes in *Vibrio* community structure rather than with increased overall abundance. Because WSSV and AHPND-causing bacteria were not detected together in the absence of BP in this observational dataset, whether BP facilitates subsequent pathogen establishment or simply co-occurs under shared predisposing conditions cannot be determined. Controlled challenge experiments will therefore be required to test these potential interactions.

Interestingly, WSSV was detected exclusively by molecular assays (PCR) and in microbiome sequencing datasets, without causing any clinical signs or severe pathology in the larvae. This discrepancy can be explained by the tank temperature, which remained above 30 °C throughout the experiment. Hyperthermia (≥30 °C) is well-documented to suppress WSSV gene expression and active viral replication without necessarily clearing the viral genome from the host [52–55]. However, the continued detection of WSSV DNA under these conditions, despite its comparatively low relative abundance in the metagenomic datasets, is consistent with the persistence of the viral genome in the absence of overt disease. This observation suggests that WSSV may persist under thermal conditions that restrict active replication, although the mechanisms underlying such persistence require further functional characterization [55].

NHP-associated bacteria and *Whispovirus* associated with WSSV were detected in the microbiome of both infected groups. Unexpectedly, PCR and histology did not reveal other NHP-causing bacteria or additional viral lesions in any samples. This discrepancy may be explained by differences in detection sensitivity or by a low viral load, since pathogen abundance is considered critical for the establishment of infection and disease progression [56,57].

The composition of the microbiome alone can explain a significant portion of the variation in survival, with taxa possessing beneficial traits, such as genes for growth factor biosynthesis, nutrient assimilation, or antioxidant production, that could increase shrimp resilience [24]. Our study revealed that both *Donghicola* and *Muricauda* were significantly more abundant in non-infected post-larvae. *Donghicola* has previously been reported to play a protective role in the microbiome of uninfected *P. vannamei*, with its effect most pronounced when combined with *Bacillus* strains [58,59]. *Muricauda*, in turn, encodes genes involved in the synthesis of antioxidants, including mannitol, glutathione, dimethylsulfide, dimethylsulfoniopropionate, zeaxanthin, and β-carotene [60]. Moreover, *Muricauda* forms consortia with microalgae that exhibit anti-*Vibrio* activity, particularly against AHPND-causing bacteria [61]. These bacteria, which produce antioxidants, may contribute to water quality and the resilience of aquatic systems under stressful conditions [62].

Metagenomic profiling showed that *Baculovirus* dominated the viral community in infected samples; concurrently, these groups exhibited a significantly higher diversity and abundance of virulence factor genes (VFGs). This enrichment pattern resembles that observed in *Macrobrachium rosenbergii* infected with *Decapod iridescent virus 1*, where genes associated with replication, recombination, and repair were also overrepresented [63]. Such functional enrichment is consistent with the concept of dysbiosis, in which disease progression is accompanied by a loss of microbial diversity and an overrepresentation of pathogenic taxa [64,65]. The enrichment of diverse KO functions found in the non-infected group highlights the presence of metabolically and functionally versatile microbial communities, supporting the validity of their classification as healthy controls in this study. Functions associated with signaling, transport, DNA repair, and mitochondrial biogenesis indicate active cellular processes and resilience to environmental or physiological stress in *Penaeus monodon* [66]. Therefore, the sharp reduction in the diversity and number of detectable KO functions in infected groups reflect a collapse of the microbial community and a limitation in functional annotation due to viral infection.

The bacterial taxa identified in infected samples were closely related to known pathogens, suggesting that infection altered not only the taxonomic composition but also the functional potential of the post-larvae microbiome, particularly through virulence-related functions [67]. Although BP infection alone was not associated with high mortality, as indicated by relatively high larval survival, mixed infection tanks containing BP, WSSV and AHPND-causing bacteria showed significantly lower survival rates, highlighting an association between multi-pathogen presence and lower survival.

The genomic architecture of nudiviruses generally encompasses 97–230 kbp. These viral sequences are commonly assembled from the sequencing data of host invertebrates, particularly through metagenomic and transcriptomic profiling [68,69]. In our study, a bin representing the most abundant *Baculovirus penaei* was recovered and annotated from the larval metagenomic samples. The near-complete consensus BP-GR25 genome is highly similar to *Penaeus vannamei nudivirus* (GenBank accession OM066077 [4],), with an average nucleotide identity (ANI) of approximately 99.5%, indicating that both genomes belong to the same nudivirus cluster. Minor differences were observed in the total genome length (ours: 119,245 bp; OM066077: 119,883 bp) and in the number of predicted ORFs (ours: 122 ORFs / 91 CDSs; [4]: 101 ORFs). The *polyhedrin* gene was also identified in our assembly, showing high sequence identity with ORF96 (*polyhedrin*) from OM066077 (positions 112,402–113,694; 1,293 nt). Both genomes share A/T-rich regulatory motifs between the transcription start site and the start codon, which could act as burst sequences analogous to those found in baculoviruses, although their regulatory role remains to be experimentally confirmed. The high ANI values, the presence of conserved core nudiviral genes, and their consistent phylogenetic clustering support the conclusion that both assemblies represent the same species-level lineage within *Gammanudivirus*.

Histology remains a valuable tool for detecting BP-induced lesions. In contrast, molecular methods, such as PCR, are preferred for detecting WSSV and AHPND-causing bacteria due to their consistent diagnostic performance. In this study, both techniques were employed in a complementary manner for group selection. Although logistical constraints prevented analysis of the same individuals using both methods, we aimed to simultaneously detect WSSV, AHPND, and BP via PCR in the exact samples designated for microbiome analysis to ensure consistency in microbial profiles. However, while PCR effectively identified WSSV and AHPND, it showed limited detection of BP, a diagnostic challenge previously reported in the literature [19,70]. Following the assembly of the near-complete BP genome in our study, in silico alignment identified a potential molecular factor contributing to this discrepancy: while the BPA primer shared 100% identity, the remaining primers (BPB through BPG) showed no significant similarity to the sequenced strain. Nevertheless, primer mismatch should not be considered the sole possible explanation for the observed histology–PCR discordance. In addition, because histological and molecular analyses were not performed on the same individuals, heterogeneous BP distribution among larvae within a tank may also have contributed to the differences between the two diagnostic approaches. To further support the histological classification used in our study, we specifically evaluated samples in which characteristic tetrahedral occlusion bodies had been observed and independently mapped metagenomic reads against the BP-GR25 genome. This comparison provided additional molecular support for BP presence in histologically compatible samples. Overall, the observed histology-PCR discordance highlights the methodological challenges of BP detection and the needed for improved molecular tools to ensure accurate pathogen correlation in future larval microbiome studies.

## Conclusions

To the best of our knowledge, this is the first study to characterize the microbiome of *P. vannamei* naturally infected with BP and to examine its interaction with WSSV and bacterial pathogens associated with AHPND. Our results revealed that BP infection was associated with a marked reduction in the richness and diversity of the postlarval microbiome. Moreover, BP-infected and mixed-infection samples exhibited broadly similar community structures that differed from the non-infected group, together with an increase in functional and virulence-related genes, which suggests a potential link to altered viral dynamics under these co-infection conditions. Although BP was the most abundant viral genus across infected groups, mortality was observed only under mixed infection conditions, suggesting that disease outcomes are closely associated with complex pathogen co-occurrence and microbiome disruption rather than with BP alone. Notably, WSSV remained detectable in mixed-infection microbiomes, despite the absence of overt clinical signs, highlighting a pattern consistent with temperature-mediated covert latency under commercial hatchery conditions (>30 °C). Understanding these microbial associations offers valuable insights into the variability in disease outcomes and provides a foundation for the development of integrated management strategies to promote microbiome stability and shrimp resilience, subject to experimental validation.

## Supporting information

S1 Table Survival and environmental/production parameters for *Penaeus vannamei* postlarvae (PL10) naturally infected by BP or mixed infection (BP + WSSV + AHPND-causing bacteria). Percentages of survival correspond to the observed postlarval populations in each tank (n = 11). Environmental variables (dissolved oxygen, temperature, salinity, and pH) and production variables (stocking density) are presented per tank. Tanks were assigned to status groups following histological evaluation.
(XLSX)

S2 Table Summary of sequencing reads, quality control, host-DNA removal, and microbial sequencing depth for each sample. (XLSX)

S3 Table Prevalence of histopathological signs associated with BP and AHPND in *Penaeus vannamei* postlarvae (PL10) naturally infected with BP alone or with mixed infections (BP + WSSV + AHPND-causing bacteria). (XLSX)

S4 Table Survival of *Penaeus vannamei* postlarvae 10 naturally infected by BP or by mixed infection (BP + WSSV + AHPND-causing bacteria). Survival percentages correspond to the observed postlarvae populations in each tank, and the presence of pathogens was confirmed by PCR and histological analysis.Tanks were reorganized for optimal grouping after evaluation of histological results. The same samples (macerated tissue) were used for microbiome analyses. The primer sets evaluated (BPA/BPB, BPA/BPG, BPD/BPB, BPD/BPG, and BPE/BPG) failed to produce any amplification products across all tested samples; furthermore, in silico analysis confirmed a lack of sequence complementarity with the *Baculovirus penaei* (BP) genome targeted in this study.
(XLSX)

S5 Table Identified open reading frames (ORFs) in the genome of *Baculovirus penaei* or *Penaeus vannamei* nudivirus BP-GR25, obtained from the VAPiD pipeline and a manual search in BLAST-NCBI. (XLSX)

S6 Table Per-sample validation of the BP-GR25 genome by independent read mapping. Host-depleted paired-end metagenomic reads from each sample were independently mapped against the 119,245-bp BP-GR25 reference genome using Bowtie2. BAM files were processed using SAMtools to determine the number of mapped reads, mapping rate, genome breadth (percentage of BP-GR25 positions covered by at least one read and mean sequencing depth. Samples 6 and 7 independently covered 100% of the BP-GR25 genome at mean depths of 81.6× and 55.2 × , respectively, while 8 covered 99.998% at 32.1 × . No high-quality SNPs were detected in these high-coverage samples. SNP variation was not assessed (NA) in samples with insufficient BP-GR25 genome coverage for reliable variant calling.
(XLSX)
