# Supplementary material for: Microbial community structure of Penaeus vannamei post-larvae naturally infected with multiple viruses in hatchery tanks
Source: PLoS One. 2026 Oct 27;21(10):e0361576. doi: 10.1371/journal.pone.0361576 (PMC13712688; doi:10.1371/journal.pone.0361576)
Supplement: Supplementary file 1 [file pone.0361576.s008.pdf]

Responses to reviewers

Submission ID PONE-D-26-29303

Editor

Editor point #1: Observational design and causal interpretation
This study is based on an observational comparison of commercial production tanks with an unbalanced number of replicates among groups. Therefore, the current data support associations between infection status, microbiome composition, and survival, but do not establish causality or pathogen synergism. Statements indicating that mixed infection "drives" mortality, that BP predisposes larvae to secondary infection, that synergistic pathogenicity was demonstrated, or that BP replication is enhanced should be substantially revised throughout the Abstract, Results, Discussion, Highlights, and Conclusions. These observations may be presented as hypotheses that require validation through controlled infection experiments.
Author response #1: We thank the reviewer for this critical observation regarding the limitations of our observational study design. We fully agree that data derived from commercial production tanks with unbalanced replication cannot establish direct causality, mechanisms of infection, or proven synergism, but rather highlight robust associations and hypotheses that require future validation through controlled challenge experiments. To address this concern comprehensively, we have thoroughly revised the manuscript across all sections (Abstract, Results, Discussion, Highlights, and Conclusions). Specifically, we have removed all causal, mechanistic, or strong definitive verbs (such as drives, predisposes, triggered by, enhanced, and synergistic pathogenic effect) and replaced them with precise, association-based terminology (such as associated with, co-occurred, combined impact, and patterns consistent with).

We also took into account the unbalanced number of samples among groups. The BP-infection group was therefore reduced from 11 to 4 samples. Importantly, as further addressed in response to Editor point #4 below, the excluded tanks showed only a very low number of reads mapping to BP-GR25, indicating limited molecular support for substantial BP presence. Consequently, all corresponding figures, abundance estimates, and statistical analyses throughout the manuscript were re-evaluated using a total of 11 samples: 4 BP-infected, 4 mixed-infection, and 3 non-infected samples. This adjustment produced only slight changes in relative abundance patterns, without altering the overall trends or main conclusions of the study.

Below is a summary of the key revisions made throughout the text:

Line	Previous manuscript	Line	Revised manuscript (track changes)
12-36	**In the Abstract section:**<br>**Background**<br>This study offers an integrative view of how viral and bacterial infections shape the microbiome and survival of *Penaeus vannamei* post-larvae, one of the most economically important species in global aquaculture.<br><br>**Results**<br>Although BP was the most abundant viral genus across infected groups, severe mortality was observed only under mixed-infection conditions, driven by *Vibrio* pathogenesis and microbiome disruption rather than by BP alone.<br><br>**Conclusion**<br>These results highlight the value of high-throughput sequencing in the early detection of pathogens in aquaculture, where subtle microbial shifts during postlarval stages can have major consequences for production.	12-43	**In the Abstract section:**<br>**Background**<br>This study offers an observational evaluation of how viral and bacterial infections co-occur with alterations in the microbiome and survival of *Penaeus vannamei* post-larvae, one of the most economically important species in global aquaculture.<br>**Results**<br>Although BP was the most abundant viral genus across infected groups, severe mortality was observed only under mixed-infection conditions, which were closely associated with AHPND-causing bacteria presence and microbiome disruption rather than with BP alone.<br>**Conclusion**<br>These results highlight the value of high-throughput sequencing in the early detection of pathogens in aquaculture, where subtle microbial shifts during postlarval stages can be valuable indicators of health status for production.
39-40	**In the Highlights section**<br>Although BP was the most abundant viral genus, mortality was observed only under mixed infections, driven by *Vibrio* pathogenesis and microbiome disruption.	52-53	**In the Highlights section**<br>Although BP was the most abundant viral genus, mortality was observed only under mixed infections.
265-266	**In the Discussion section**<br>High mortality was restricted to mixed-infection tanks, indicating that BP alone does not drive mortality under these conditions. Instead, BP may predispose larvae to secondary pathogens.	334-339	**In the Discussion section**<br>High mortality was restricted to mixed-infection tanks, indicating that BP alone was not linked to high mortality under these conditions. Although BP infection alone was associated with the lowest *Vibrio* abundance, Vibrio community composition differed significantly between BP-infected and non-infected groups, suggesting that BP may be associated with changes in Vibrio community structure rather than with increased overall abundance.

308-311	Although BP infection alone did not lead to high mortality, as indicated by relatively good larval survival, our findings show that co-infection with WSSV and AHPND-causing bacteria significantly reduced survival rates, highlighting a synergistic pathogenic effect.	338-392	Although BP infection alone was not associated with high mortality, as indicated by relatively high larval survival, mixed infection tanks containing BP, WSSV and AHPND-causing bacteria showed significantly lower survival rates, highlighting an association between multi-pathogen presence and lower survival.
340-352	**In the Conclusions section**<br>To the best of our knowledge, this is the first study to characterize the microbiome of *P. vannamei* naturally infected with BP and to examine its interaction with WSSV and bacterial pathogens associated with AHPND. Our results revealed that BP infection markedly reduced the richness and diversity of the postlarval microbiome. Moreover, microbiome composition differed substantially between samples with BP alone and those with mixed infection, with an increase in functional and virulence-related genes, suggesting that BP replication may be enhanced under these conditions. Although BP was the most abundant viral genus across infected groups, mortality was observed only under mixed infection conditions, suggesting that disease outcomes may be driven by pathogen interactions and microbiome disruption rather than by BP alone. Notably, high WSSV abundance was detected in mixed-infection microbiomes despite the complete absence of clinical signs, indicating a temperature-mediated covert latency triggered by commercial hatchery conditions (>30 °C). Understanding these microbial interactions offers valuable insights into the variability in disease outcomes and supports the development of integrated management strategies to promote microbiome stability and shrimp resilience.	431-447	**In the Conclusions section**<br>To the best of our knowledge, this is the first study to characterize the microbiome of *P. vannamei* naturally infected with BP and to examine its interaction with WSSV and bacterial pathogens associated with AHPND. Our results revealed that BP infection was associated with a marked reduction in the richness and diversity of the postlarval microbiome. Moreover, BP-infected and mixed-infection samples exhibited broadly similar community structures that differed from the non-infected group, together with an increase in functional and virulence-related genes, which suggests a potential link to altered viral dynamics under these co-infection conditions. Although BP was the most abundant viral genus across infected groups, mortality was observed only under mixed infection conditions, suggesting that disease outcomes are closely associated with complex pathogen co-occurrence and microbiome disruption rather than with BP alone. Notably, WSSV remained detectable in mixed-infection microbiomes, despite the absence of overt clinical signs, highlighting a pattern consistent with temperature-mediated covert latency under commercial hatchery conditions (>30 °C). Understanding these microbial associations offers valuable insights into the variability in disease outcomes and provides a foundation for the development of integrated management strategies to promote microbiome stability and shrimp resilience, subject to experimental validation.

Editor point #2: Experimental unit, tank-level metadata, and possible confounding variables

Please explicitly state that the tank is the experimental/statistical unit, whereas the post-larvae analyzed from each tank represent a pooled biological sample. The approximate number of post-larvae contributing to each ~1 g sample should also be reported.

In addition, please provide a comprehensive supplementary table containing, for each of the 18 tanks, survival, infection status, sampling information, and all available environmental/production variables, including temperature, salinity, pH, dissolved oxygen, stocking density, water source, and any relevant management practices. These variables should be statistically compared among groups whenever the data are available.

Author response #2:

We thank the editor and reviewers for these comments. The manuscript has been revised as follows: The Materials and Methods section now explicitly states that the tank is the statistical unit ($n = 11$). We also clarified that PLs per tank represent a pooled biological sample of approximately 250 postlarvae per ~1 g sample. Additionally, we have added Supplementary Table 1 detailing individual metadata for all 11 tanks, including survival (%), health status, sampling info, stocking density, water source, routine management, and values for dissolved oxygen, temperature, salinity, and pH. ANOVA confirmed no significant differences among health status groups for any environmental variable ($p > 0.05$), except for survival, which has been detailed in the Results section under "Sample collection, diagnosis, and classification of infection status". The main changes are presented in the table below:

Line	Previous manuscript	Line	Revised manuscript (track changes)
92-100	**In the Materials and Methods section**<br>**Sample collection, diagnosis, and classification of infection status**<br>To capture a representative snapshot of the system, *Penaeus vannamei* post-larvae (PL10 stage) were sampled in parallel from 18 commercial-scale production tanks, each possessing a 35-ton capacity. The collection was carried out at a commercial hatchery facility in South America with a well-documented record of persistent viral outbreaks. All tanks were managed under the same feeding protocol throughout the production cycle. Each sample was divided into two subsamples for distinct analyses: (1) approximately 1 g of randomly collected larvae was fixed in Davidson's solution and processed for histopathological examination, and (2) approximately 1 g of larvae was macerated and preserved in DNA/RNA Shield solution at −20 °C for subsequent pathogen PCR diagnostics and HTS-based microbiome analysis.	101-116	**In the Materials and Methods section**<br>**Sample collection, diagnosis, and classification of infection status**<br>To capture a representative snapshot of the system and considering the individual tank as the experimental and statistical unit, *Penaeus vannamei* post-larvae (PL10 stage) were sampled in parallel from 11 commercial-scale production tanks, each possessing a 35-ton capacity. The collection was carried out at a commercial hatchery facility in South America with a well-documented record of persistent viral outbreaks. All tanks were managed under the same feeding protocol throughout the production cycle. For each tank, the post-larvae analyzed represented a pooled biological sample. Each sample was divided into two subsamples for distinct analyses: (1) approximately 1 g of randomly collected larvae (representing approximately 250 individuals) was fixed in Davidson's solution and processed for histopathological examination, and (2) approximately 1

			g of larvae was macerated and preserved in DNA/RNA Shield solution at −20 °C for subsequent pathogen PCR diagnostics and HTS-based microbiome analysis.<br>Environmental and production variables across the 11 tanks (detailed in Supplementary Table 1) were statistically compared among groups to assess potential confounding effects. Data were analyzed using analysis of variance (ANOVA) after verifying normality (Shapiro-Wilk test) and homoscedasticity (Levene's test). Differences among group means were identified using Tukey's honest significant difference (HSD) post-hoc test.
		220-226	**In the Results section**<br>**Sample collection, diagnosis, and classification of infection status**<br>At the PL11 harvest, survival differed significantly among groups, with mixed-infections tanks showing the lowest survival (27.5 ± 1.1%), followed by BP-infected tanks (63.3 ± 2.2%), and non-infected tanks (70.8 ± 0.9%). Tukey's HSD post-hoc test confirmed significant differences for all pairwise comparisons ($p < 0.001$). In contrast, no significant differences were detected among groups for dissolved oxygen, temperature, salinity, and pH (ANOVA, $p > 0.05$), indicating that the monitored environmental parameters did not differ significantly among infection groups.

Editor point #3: Host DNA removal and effective microbial sequencing depth

The manuscript states that host-derived contigs were removed, but the host-depletion strategy is insufficiently described. Please provide full methodological details, including the reference genome used, filtering criteria, whether filtering was performed at the read or contig level, and the software/parameters employed. It is necessary to report the number of final reads used for the assembly and the calculation of the abundances compared to the initial reads, and the number of reads that mapped to shirmp and human genomes.

Importantly, provide per-sample statistics including raw reads, reads retained after quality control, host-associated reads, microbial reads, viral reads where applicable, mapped reads, and mapping percentages.

Author response #3: We thank the editor and reviewer for highlighting the need for a more detailed description of our host-depletion strategy and sequencing statistics. We have revised the Materials and Methods section to provide full methodological details regarding host DNA removal. Host-depletion was performed at the read level prior to assembly using KneadData integrated with Bowtie2. Reads were filtered against the *Penaeus vannamei* reference genome. Therefore, we have added Supplementary Table 2, which provides the complete per-scale and per-sample statistics requested.

The changes are detailed on the table below

Line	Previous manuscript	Line	Revised manuscript (track changes)
128-130	**In the Material and Methods section**<br>**Metagenome assembly and annotation**<br>Metagenomic reads were processed using the SqueezeMeta pipeline (v1.7.2) [40].	145-149	**In the Material and Methods section**<br>**Metagenome assembly and annotation**<br>Prior to assembly, host-derived reads were systematically depleted using Kneaddata (v0.12.0; https://github.com/biobakery/kneaddata) with a custom database containing the *P. vannamei* genome (NCBI RefSeq Assembly ID: GCA_042767895.1) via Bowtie2 (--very-sensitive mode). Per-sample read statistics across processing steps are provided in Supplementary Table 2. Retained host-depleted metagenomic reads were processed using the SqueezeMeta pipeline (v1.7.2).

Editor point #4: Validation of the BP-GR25 genome and the claim of genome completeness

The manuscript refers to BP-GR25 as a "complete genome"; however, the evidence currently presented is insufficient to establish completeness. In particular, CheckM is primarily designed for bacterial and archaeal genome quality assessment and cannot by itself validate the completeness of a nudivirus genome. Please provide virus-appropriate evidence supporting genome completeness, including the number of contigs comprising BP-GR25, coverage across the genome, assembly-graph or junction evidence where applicable, and the presence of expected conserved nudiviral core genes.

If genome closure or completeness cannot be conclusively demonstrated, please use a more conservative designation such as "near-complete genome" or "metagenome-assembled consensus genome."

Author response #4: We sincerely appreciate the reviewer's valuable feedback regarding the limitations of CheckM for viral genome assessment. We completely agree that CheckM is tailored for bacteria and archaea and is insufficient for validating the nudivirus genome. In response to this comment, we have re-analyzed the assembly using CheckV, the current gold-standard tool specifically designed for assessing the

quality and completeness of viral genomes recovered from metagenomes. The CheckV analysis classified the *Baculovirus penaei* BP-GR25 assembly as a High-quality viral genome with an estimated completeness of 96.13%. Furthermore, we have revised the manuscript throughout to adopt the conservative and accurate designation "near-complete consensus genome" instead of "complete genome".

A summary of these changes across the manuscript sections is provided below:

Line	Previous manuscript	Line	Revised manuscript (track changes)
30-31	**In the Abstract section**<br>**Results**<br>The complete BP genome (119,245 bp) was assembled and annotated from larval metagenomes, revealing conserved core nudiviral genes and consistent species-level phylogenetic clustering.	37-38	**In the Abstract section**<br>**Results**<br>The near-complete consensus BP-GR25 genome (119,245 bp) was assembled and annotated from larval metagenomes, revealing conserved core nudiviral genes and consistent species-level phylogenetic clustering.
164-165	**In the Material and Methods section**<br>***Baculovirus penaei* genome assembly**<br>Through binning with DAS Tool (v1.1.7), the genome of *Baculovirus penaei* BP-GR25 was recovered from larval microbiome samples. Genome quality was evaluated with CheckM (v1.1.6) [47],	186-187	**In the Material and Methods section**<br>***Baculovirus penaei* genome assembly**<br>Through binning with DAS Tool (v1.1.7), the genome of *Baculovirus penaei* BP-GR25 was recovered from larval microbiome samples. Genome quality was evaluated with CheckV (v1.1.1) [46],
315-316	**In the discussion section**<br>The complete BP genome is highly similar to *Penaeus vannamei* nudivirus (GenBank accession OM066077, [4]),	396-397	**In the discussion section**<br>The near-complete consensus BP-GR25 genome is highly similar to *Penaeus vannamei* nudivirus (GenBank accession OM066077, [4]),
333-334	Following the assembly of the complete BP genome in our study, in silico alignment provided a molecular explanation for this discrepancy:	415-416	Following the assembly of the near-complete BP genome in our study, in silico alignment identified a potential molecular factor contributing to this discrepancy

Editor point #5: Per-sample validation and strain variation of BP-GR25
Because BP-GR25 was recovered from a co-assembly, please determine whether the assembled sequence represents a single strain or a consensus derived from genetically distinct BP populations across tanks. Reads from each BP-positive and mixed-infection sample should be independently mapped against BP-GR25. Please provide, for each sample, breadth and depth of coverage and, where possible, an assessment of sequence variation/SNP density. This analysis is also important to exclude possible assembly artifacts or chimeric regions and to evaluate strain-level variation among tanks.
Author response #5: We sincerely thank the Editor for this highly valuable comment. This analysis provided an important methodological perspective for our study and will be very useful in our future metagenomic investigations. Independently remapping reads from positive samples against recovered viral or bacterial genomes provides a practical approach to evaluate genome reconstruction, coverage breadth and depth, and potential strain-level variation through SNP analysis.
In response to this comment, we independently mapped each sample against BP-GR25 and calculated mapped reads, mapping rate, genome breadth, mean depth, and SNP density. We also compared these results with the metagenomic abundance patterns to evaluate their consistency. The corresponding methods, results, and per-sample statistics have now been incorporated into the revised manuscript and Supplementary Table 6. We greatly appreciate this suggestion, as it strengthened the validation of BP-GR25 and provided a valuable approach for our future studies.

Line	Previous manuscript	Line	Revised manuscript (track changes)
163-172	**In the Material and Methods section**<br>***Baculovirus penaei* genome assembly**<br>Through binning with DAS Tool (v1.1.7), the genome of *Baculovirus penaei* BP-GR25 was recovered from larval microbiome samples. Genome quality was evaluated with CheckM (v1.1.6) [47], while annotation and identification were performed using the VAPiD pipeline (v1.2) [48], complemented by a manual search for coding regions in BLAST-NCBI [49]. To evaluate sequence similarity at the whole-genome level, an Average Nucleotide Identity (ANI) analysis was executed via fastANI (v1.34). The genomic architecture was visualized as a circular map using the Genovi pipeline (v0.2.16) [50]. Furthermore, evolutionary relationships were investigated through a genome-scale phylogenomic analysis executed within the PhyloSuite platform (v2.0) [51]. Public access to the baculovirus BP-GR25 genome is provided via the	186-204	**In the Material and Methods section**<br>***Baculovirus penaei* genome assembly**<br>Through binning with DAS Tool (v1.1.7), the genome of *Baculovirus penaei* BP-GR25 was recovered from larval microbiome samples. Genome quality was evaluated with CheckVM (v1.1.16) [46], while annotation and identification were performed using the VAPiD pipeline (v1.2) [47], complemented by a manual search for coding regions in BLAST-NCBI [48]. To evaluate sequence similarity at the whole-genome level, an Average Nucleotide Identity (ANI) analysis was executed via fastANI (v1.34). The genomic architecture was visualized as a circular map using the Genovi pipeline (v0.2.16) [49]. Furthermore, evolutionary relationships were investigated through a genome-scale phylogenomic analysis executed within the PhyloSuite platform (v2.0) [50]. Public access to the near-complete BP-GR25 genome is provided via the

	GenBank repository, where it was registered under the specific accession number PZ441674.		GenBank repository, where it was registered under the specific accession number PZ441674. To independently validate the BP-GR25 genome across samples and assess potential strain-level variation, host-depleted paired-end metagenomic reads from each sample were independently mapped against the 119,245-bp BP-GR25 reference genome using Bowtie2 (v2.5.4) with the --very-sensitive alignment preset. The resulting alignments were converted to BAM format, sorted, and indexed using SAMtools (v1.24). Per-sample mapping statistics were calculated, including the number of mapped reads, mapping rate, genome breadth (percentage of reference positions covered by at least one read), mean and sequencing depth. Sequence variation relative to BP-GR25 was assessed using BCFtools (v1.24) through pileup generation and variant calling. High-quality SNPs were retained using minimum thresholds of read depth ≥10 and variant quality (QUAL) ≥30, and SNP density was calculated relative to the BP-GR25 genome length.
246-253	**In the Results section**<br>***Baculovirus penaei* genome**<br>The *Baculovirus penaei* BP-GR25 genome is 119,245 bp in length, with a GC content of 33.3% and an average read coverage of 42x (Data set 2). Homology-based gene prediction identified a total of 122 ORFs, of which 91 were CDSs (Fig 5A, Supplementary Table 3). The genome possesses high COG abundance in lipid transport and metabolism, signal transduction, intracellular trafficking, secretion, and vesicular transport, replication, recombination, and repair, among other unknown functions (Fig 5B). The genome showed average nucleotide identity (ANI) values of 99.45% with Penaeus vannamei nudivirus (GenBank accession OM066077.1) and was clustered within Penaeus monodon nudivirus (Fig 5C).	303-319	**In the Results section**<br>***Baculovirus penaei* genome assembly**<br>The *Baculovirus penaei* BP-GR25 genome is 119,245 bp in length, with a GC content of 33.3% and an average read coverage of 42x. Homology-based gene prediction identified a total of 122 ORFs, of which 91 were CDSs (Fig 5A, Supplementary Table 53). CheckV analysis validated the assembly as a High-quality viral genome with an estimated completeness of 96.13%. Independent mapping of host-depleted reads from each tank against BP-GR25 further supported the integrity of the recovered genome. Two samples from the mixed-infection group independently covered 100% of the 119,245-bp BP-GR25 genome, with mean sequencing depths ranging from 55.2× to 81.6×, while a third mixed-infection sample showed 99.99% genome breadth at a mean depth of 32.1× (Supplementary Table 6). No high-quality SNPs were detected in samples with sufficient genome coverage for reliable variant assessment. The near-complete to complete genome-wide coverage obtained independently from these samples, together with the absence of detectable sequence variation, supports BP-GR25 as a coherent and highly conserved viral genome rather than a chimeric sequence resulting from the co-assembly. The genome possesses high COG abundance in lipid transport and metabolism, signal transduction, intracellular trafficking, secretion, and vesicular transport, replication, recombination, and repair, among other unknown functions (Fig 5B). The genome showed average nucleotide identity (ANI) values of 99.45% *with Penaeus vannamei nudivirus* (GenBank accession OM066077.1) and was clustered within *Penaeus monodon nudivirus* (Fig 5C).

Editor point #6: Identification of AHPND-associated bacteria and interpretation of Vibrio
Detection of the PirAB-associated AP4 target establishes the presence of AHPND-associated genetic determinants but does not necessarily establish *Vibrio parahaemolyticus* as the carrier, since AHPND can be associated with different Vibrio species. If the metagenomic data permit, please determine the taxonomic identity of the PirAB-carrying organism using appropriate genomic or marker-based evidence. Otherwise, the manuscript should consistently use terminology such as "AHPND-causing bacteria" and avoid attributing the phenotype specifically to *V. parahaemolyticus* without direct evidence.
Author response #6: Thank you for this important clarification. We agree that detection of the PirAB-associated AP4 target does not provide direct evidence that *Vibrio parahaemolyticus* is the carrier of the AHPND-associated genetic determinants. Accordingly, throughout the manuscript, we have revised the terminology to consistently use "AHPND-causing bacteria" and have avoided specifically attributing the AHPND phenotype to *V. parahaemolyticus* in the absence of direct evidence identifying the PirAB-carrying organism.

Reviewer 1

Reviewer 1 point #1: Please add geographic location of the sampling sites, this will help readers to see distribution of the 18 field sites.
Author response #1: The study was performed in Santa Elena, Ecuador. However, the Ecuadorian competent authorities have not reported to the World Organization for Animal Health (OIE) the AHPND presence in the territory. For this reason, we cannot report that the study was performed in Ecuador. In this context, we prefer to mention the region and not the country. We hope the editors and reviewers understand this sensitive situation and allow us to publish that the study was performed in South America.

Reviewer 1 point #2: The study compares 11 BP-positive tanks, four mixed-infection tanks, and three non-infected tanks, which is quite interesting. However, the proportion in the mixed group ,containing BP, WSSV and AHPND, could not be quantitatively measured, along with potential tank-effect as an additional factor. Therefore, statements that mixed infection “drives” mortality, BP predisposes larvae to secondary infection, or synergistic pathogenicity was demonstrated should be revised perhaps, softened and presented as hypotheses for a future controlled challenge trial.
Author response #2: We thank the reviewer for this observation. This concern was also raised in Editor Point #1 and has been addressed comprehensively throughout the revised manuscript. We agree that the unbalanced observational design and potential tank-level effects preclude causal inference or demonstration of pathogen synergism. Accordingly, causal statements were replaced with association-based terminology, and potential pathogen interactions are now presented as hypotheses requiring validation through controlled challenge experiments.

Reviewer 1 point #3: Please add % survival of each individual tank from each group (Table 1), and define whether reported variation was SD or SEM values (Table 1). The authors should also perform statistical comparison of %survival. It’s also very important to provide water parameters of each tank and also stat tests of each key parameter to explore whether environmental factors play roles in different survival levels or not.
Author response #3: We thank the reviewer for this recommendation. As also described in our response to the Editor, a new Supplementary Table 1 has been added to the revised manuscript, reporting the survival percentage and environmental/production parameters for each individual tank. Group-level variation is reported as mean ± SD, as specified in the table. Survival percentages were statistically compared among infection groups using ANOVA followed by Tukey’s HSD post-hoc test. In addition, dissolved oxygen, temperature, salinity, and pH were statistically compared among groups to assess potential differences in environmental conditions. The corresponding results have been added to the “Sample collection, diagnosis, and classification of infection status” section. At the PL11 harvest, survival differed significantly among groups, with mixed-infection tanks showing the lowest survival (27.5 ± 1.1%), followed by BP-infected tanks (63.3 ± 2.2%) and non-infected tanks (70.8 ± 0.9%). Tukey’s HSD post-hoc test confirmed significant differences for all pairwise comparisons ($p < 0.001$). In contrast, no significant differences were detected among groups for dissolved oxygen, temperature, salinity, or pH (ANOVA, $p > 0.05$).

Reviewer 1 point #4: Please verify the total read yield and per-sample average: the manuscript states 1,498 million clean reads across the 18 samples, “averaging 74 million reads per sample”, but 1,498 / 18 ≈ 83 million. Please correct whichever figure is inaccurate.
Author response #4: Thank you for pointing out this inconsistency. We rechecked the sequencing data for all 11 new samples and corrected the corresponding values in the manuscript.
Therefore, here you can check the changes:

Line	Previous manuscript	Line	Revised manuscript (track changes)
191-199	**In the Results section**<br>**Metagenome assembly and annotation**<br>Metagenomic sequencing yielded a total of 1,498 million clean reads across the 18 *P. vannamei* larval samples, averaging 74 million reads per sample. Following co-assembly, 9,8 million contigs were generated (longest contig: 1,184,601 bp). Gene prediction identified an average of 4,9 million ORFs per sample. Most ORFs (>99%) were detected by Prodigal, while Aragorn and	232-245	**In the Results section**<br>**Metagenome assembly and annotation**<br>Metagenomic sequencing yielded a total of 732 million clean reads across the 11 *P. vannamei* larval samples, averaging 67 million reads per sample. Following co-assembly, 2,9 million contigs were generated (longest contig: 523,334 bp). Gene prediction identified 3,433,465 protein-coding ORFs using Prodigal. In addition to protein-coding genes, RNA gene prediction identified 11,504

	Barrnap contributed only minor fractions corresponding to tRNA/tmRNA (≈33,000 per sample) and rRNA genes (≈1,000 per sample), respectively. Regarding functional databases, KEGG orthologs were assigned to an average of approximately 79,600 genes per sample, while COG annotations accounted for ~116,000 genes per sample. Pfam domains were detected in ~60,000 ORFs.		tRNA/tmRNA sequences with Aragorn and 933 rRNA genes with Barrnap. Regarding functional databases, 584,010 ORFs (17.0% of the predicted ORFs) received KEGG functional assignments, representing 11,793 KEGG entries, whereas 849,354 ORFs (24.7%) were assigned to COG categories, encompassing 35,393 COG entries. Genome-resolved reconstruction recovered 108 metagenome-assembled genomes (MAGs), of which 15 met high-quality criteria (>90% completeness and <10% contamination) and 25 met good-quality criteria (>75% completeness and <10% contamination).

Reviewer 1 point #5: The sequencing platform is reported inconsistently: the Abstract states microbiome profiling was done "on the Illumina NovaSeq X plus platform," while the Methods section ("DNA extraction and microbiome sequencing") states sequencing "was performed on an Illumina NovaSeq 6000 platform." Please confirm which instrument was actually used and correct throughout.

Author response #5: Thank you for identifying this inconsistency. We verified the sequencing platform used in this study and have corrected the corresponding information throughout the manuscript to ensure consistency.

Reviewer 1 point #6: In Fig. 4, infected samples show COG enrichment for retrotransposon proteins, transposases, and reverse transcriptases, please discuss whether these genes encoded by baculoviruses or nudiviruses or microbes. Or do they reflect residual host (shrimp) genomic DNA contamination in the metagenome? Please clarify this point explicitly, as distinguishing between host contamination and bacterial dysbiosis substantially alters the biological interpretation.

Author response #6: Thank you for raising this important point. We agree that distinguishing host-derived sequences from microbial mobile genetic elements is essential for the biological interpretation of these results. We therefore clarified this aspect in the revised manuscript. Host-associated reads were removed prior to metagenomic assembly by mapping the quality-filtered reads against the shrimp reference genome. Consequently, the functional analysis shown in Fig. 4 was performed on the host-depleted metagenomic dataset. The COG categories enriched in infected samples, including retrotransposon proteins, transposases, reverse transcriptases, integrase-related proteins, and recombination-associated functions, are not specific to baculoviruses or nudiviruses. These functions are also commonly associated with bacterial mobile genetic elements, including insertion sequences, transposons, integrative elements, and other mobile DNA. Based on the taxonomic evaluation of the corresponding metagenomic sequences, these signals were interpreted primarily as microbial/mobile genetic element-associated functions rather than residual shrimp genomic DNA. We have explicitly clarified this point in the revised Discussion and have moderated the interpretation to avoid attributing these functions specifically to viral genomes without direct taxonomic evidence.

Reviewer 1 point #7: Please clarify the unit of replication used for microbiome sequencing: how many post-larvae were pooled into the ~1 g subsample per tank that was preserved for HTS? This matters for interpreting whether the diversity/statistical comparisons treat tanks or pooled individuals as the sampling unit.

Author response #7: Thank you for this comment. This issue has already been addressed in our previous response to the Editor point 2. The number of post-larvae pooled to obtain the ~1 g subsample used for HTS, as well as the unit of replication used for microbiome analyses, has been clarified in the revised manuscript.

Reviewer 1 point #8: Gene abundances were normalized as "Transcripts Per Million (TPM)", but this is a shotgun DNA metagenomic dataset rather than an RNA-seq/transcriptomic one. Please clarify or correct this terminology (e.g., reads-per-million or a similarly DNA-appropriate metric) if "transcripts" was carried over from RNA-seq convention in error.

Author response #8: We thank the reviewer for pointing out this ambiguity. We have revised the manuscript to clarify the terminology and the normalization procedure used for the functional abundance analyses. The reference to "Transcripts Per Million (TPM)" resulted from a confusion in describing the SqueezeMeta/SQMtools pipeline. SqueezeMeta reports TPM as one of its available normalized abundance measures for genes/ORFs, including in shotgun metagenomic analyses. In this context, TPM does not represent experimentally measured transcript abundance, as in RNA-seq. Rather, it is a length- and

sequencing-depth-normalized metric calculated from metagenomic DNA reads mapped to predicted genes/ORFs. Thus, the use of the term TPM in the SqueezeMeta output is not indicative of transcriptomic data; however, we agree with the reviewer that referring to our functional abundances as "Transcripts Per Million" was potentially misleading in the context of a DNA shotgun metagenomic study. Importantly, TPM-normalized values were not the abundance values ultimately used for our comparative analyses. The functional count tables generated from the SqueezeMeta/SQMtools outputs were imported into microeco using the mapped metagenomic read counts. These count data were processed using the same normalization framework applied to the taxonomic abundance data: Cumulative Sum Scaling (CSS) normalization was applied prior to the downstream comparative analyses. We have therefore removed the statement that gene abundances were normalized as "Transcripts Per Million (TPM)" and revised the Methods to specify that functional abundances were represented by mapped metagenomic read counts and subsequently normalized using CSS in microeco.

Line	Previous manuscript	Line	Revised manuscript (track changes)
158-159	**In the Material and Methods section**<br>**Microbiome function and associated virulence factors**<br>Microbiome function and associated virulence factors<br>Gene prediction was performed on the assembled contigs using Prodigal (v2.6.3) for protein-coding genes, and Barrnap (v0.9) for rRNA gene detection. The resulting open reading frames (ORFs) were functionally annotated. Homology research was conducted against the GenBank nr database using DIAMOND (v2.1.14) [45] for taxonomic assignment, and against the COG and Pfam databases using HMMER (v3.4) [46]. Functional count tables were generated using the SQMtools R package based on the annotated ORFs obtained from SqueezeMeta. Gene abundances were normalized to Transcripts Per Million (TPM) to account for differences in sequencing depth and gene length.	173-181	**In the Material and Methods section**<br>**Microbiome function and associated virulence factors**<br>Gene prediction was performed on the assembled contigs using Prodigal (v2.6.3) for protein-coding genes, and Barrnap (v0.9) for rRNA gene detection. The resulting open reading frames (ORFs) were functionally annotated. Homology research was conducted against the GenBank nr database using DIAMOND (v2.1.14) [45] for taxonomic assignment, and against the COG and Pfam databases using HMMER (v3.4) [46]. Functional count tables were generated using the SQMtools R package based on the annotated ORFs obtained from SqueezeMeta. Functional abundances were represented as mapped metagenomic read counts, and the resulting functional count matrix was normalized using Cumulative Sum Scaling (CSS) prior to comparative analyses.

Reviewer 1 point #9: In Table 1, what the "2 Positive / 9 Negative (BP)" and "BP (1/4)" notations represent (i.e., PCR-positive tanks out of total tanks tested per group), since the column header lists three pathogens (WSSV/AHPND/BP) but the BP-positive row only reports one ratio.

Author response #9: Thank you for pointing out this ambiguity. We have revised Table 1 to clarify the notation used for the PCR results. The reported fractions represent the number of PCR-positive tanks out of the total number of tanks evaluated within each group. Thus, in the BP-positive group, "2 Positive / 9 Negative (BP)" indicated that BP was detected by PCR in 2 of the 11 tanks, whereas the remaining 9 tanks were PCR-negative for BP. Similarly, in the mixed-infection group, "BP (1/4)" indicated that 1 of the 4 tanks was PCR-positive for BP, while AHPND-causing bacteria and WSSV were detected in all 4 tanks (4/4). To avoid confusion, we have standardized the PCR results in Table 1 as the number of positive tanks over the total number of tanks tested (positive tanks/total tanks) and maintained the same pathogen order as indicated in the column heading (WSSV / AHPND-causing bacteria / BP).

Reviewer 1 point #10: The Discussion and Conclusion both describe WSSV as showing "high genomic abundance" despite covert/asymptomatic infection, but Fig 3B shows WSSV at a much lower relative abundance — far below Baculovirus. The authors should explain what "high" means here (e.g., high relative to non-infected, or high viral copy number despite low relative abundance in the whole metagenome) so this isn't read as contradictory.

Author response #10: Thank you for highlighting this potentially misleading wording. We agree that the term "high WSSV abundance" could be interpreted as indicating that WSSV was among the most abundant viral taxa in the metagenome, which is not supported by Fig. 3B. In fact, WSSV showed a comparatively low relative abundance, particularly in relation to *Baculovirus*, which was the predominant viral genus across infected groups. Therefore, we have modified the respective sections:

Line	Previous manuscript	Line	Revised manuscript (track changes)
27-29	**In the Abstract section**<br>**Results**<br>In contrast, *Vibrio* abundance and PirAB toxin genes increased significantly during mixed infections, whereas high WSSV genomic abundance persisted across datasets, despite the infection remaining thermally suppressed and asymptomatic in the larvae.	31-39	**In the Abstract section**<br>**Results**<br>In contrast, mixed infections were characterized by a relative increase in *Vibrio*, together with the PCR-detection of PirAB toxin genes, whereas WSSV remained detectable in the metagenomic dataset despite its comparatively low relative abundance, while the infection remained thermally suppressed and asymptomatic in the larvae.

273-276	**In the Discussion section**<br>However, the persistence of a high viral DNA payload under thermal stress suggests that the virus may utilize uncharacterized adaptation mechanisms to maintain genomic stability. Rather than a passive state of latency, this genomic stability implies a complex, host-chaperone-modulated equilibrium that warrants further functional characterization [56].	349-356	**In the Discussion section**<br>However, the continued detection of WSSV DNA under these conditions, despite its comparatively low relative abundance in the metagenomic datasets, is consistent with the persistence of the viral genome in the absence of overt disease. This observation suggests that WSSV may persist under thermal conditions that restrict active replication, although the mechanisms underlying such persistence require further functional characterization [55].
348-350	**In the Conclusions section**<br>Notably, high WSSV abundance was detected in mixed-infection microbiomes despite the complete absence of clinical signs, indicating a temperature-mediated covert latency triggered by commercial hatchery conditions (>30 °C).	441-444	**In the Conclusions section**<br>Notably, WSSV remained detectable in mixed-infection microbiomes, despite the absence of overt clinical signs, highlighting a pattern consistent with temperature-mediated covert latency under commercial hatchery conditions (>30 °C).

Reviewer 1 point #11: In the result section, 11 tanks were histologically positive for BP (tetrahedral occlusion bodies), but PCR confirmed only 2/11 (Table 1). The authors mentioned that this could be related to primer mismatch (BPB–BPG vs. the sequenced strain). However, could it also be due to the possibility that some "tetrahedral occlusion bodies" seen histologically could be a non-BP artifact, given the low PCR confirmation rate as well. Please elaborate and discuss this.

Author response #11: We thank the reviewer for this important observation. We agree that primer mismatch should not be considered the sole explanation for the histology–PCR discordance. Importantly, histological and molecular analyses were not performed on the same individual larvae; therefore, heterogeneous BP distribution among larvae within the same tank may have contributed to the low PCR confirmation rate. To avoid potential misinterpretation between the histological observations and PCR results, we further reinforced the classification by independently mapping metagenomic reads from histologically BP-compatible samples against the BP-GR25 genome. For the subsequent comparative analyses, we retained only those samples showing clear and substantial mapping support to BP-GR25. This additional molecular validation provided stronger support for BP presence in the selected samples and reduced potential inconsistencies among diagnostic approaches. The Discussion has been revised accordingly.

Reviewer 1 point #12: The BP-GR25 genome was recovered via co-assembly/binning across pooled metagenomes from multiple tanks (both BP-only and mixed-infection groups). Was strain-level variation among tanks assessed, or does BP-GR25 represent a single consensus that could be masking real strain diversity across the 15 infected tanks?

Author response #12: We sincerely thank the Reviewer for this important comment. Strain-level variation among tanks was assessed by independently mapping reads from each sample against the BP-GR25 genome and evaluating genome breadth, sequencing depth, and SNP variation. No high-quality SNPs were detected among samples with sufficient genome coverage, indicating no detectable strain-level variation and supporting the presence of a single highly conserved BP-GR25 strain, within the resolution of our analysis, rather than a consensus masking genetically distinct BP populations. Samples with insufficient coverage were not used to infer the absence of variation. The complete analysis and methodological details are provided above in our response to Editor Point #5, and the corresponding results have been incorporated into the revised manuscript and Supplementary Table 6.

Reviewer 1 point #13: The manuscript reports Vibrio as "3.2-fold more abundant in mixed infection than in other health status," can the authors explain why Vibrio was also increased in BP-only tanks relative to non-infected ones. Given the hypothesis that BP predisposes larvae to secondary bacterial infection, this specific comparison (BP-only vs. non-infected) is also important to report and discuss.

Author response #13: We thank the reviewer for this observation. We have clarified the *Vibrio* abundance comparisons in the revised manuscript. Contrary to the apparent pattern in the original presentation, *Vibrio* relative abundance was lower in BP-only than in non-infected tanks, whereas a marked relative increase was observed in mixed-infection tanks. Given this finding, we additionally analyzed *Vibrio* beta diversity

to determine whether the Vibrio-assigned taxa differed between BP-only and non-infected groups despite the lower overall abundance. PERMANOVA revealed a significant difference in *Vibrio* community composition between these groups (Bray-Curtis, adjusted p = 0.045), with no significant difference in multivariate dispersion (PERMDISP, p = 0.739). This finding is particularly interesting because it suggests that BP infection may be associated with differences in *Vibrio* community composition rather than with an increase in overall *Vibrio* abundance. Therefore, these results have now been reported and discussed in the revised manuscript.

Line	Previous manuscript	Line	Revised manuscript (track changes)
21-29	**In the abstract section**<br>**Results**<br>Although BP was the most abundant viral genus across infected groups, severe mortality was observed only under mixed-infection conditions, driven by *Vibrio* pathogenesis and microbiome disruption rather than by BP alone. Deep metagenomic sequencing revealed reduced, distinct microbiome structures in shrimp infected with BP and in those with mixed infections, compared to non-infected individuals. Non-infected post-larvae maintained complex and well-balanced bacterial communities, predominantly composed of *Donghicola* and *Muricauda*. In contrast, *Vibrio* abundance and *PirAB* toxin genes increased significantly during mixed infections, whereas high WSSV genomic abundance persisted across datasets, despite the infection remaining thermally suppressed and asymptomatic in the larvae.	25-36	**In the abstract section**<br>**Results**<br>Although BP was the most abundant viral genus across infected groups, severe mortality was observed only under mixed-infection conditions, which were closely associated with AHPND-causing bacteria presence and microbiome disruption rather than with BP alone. Deep metagenomic sequencing revealed reduced, distinct microbiome structures in shrimp infected with BP and in those with mixed infections, compared to non-infected individuals. Non-infected post-larvae maintained complex and well-balanced bacterial communities, predominantly composed of *Donghicola* and *Muricauda*. Nudiviridae and Oscillospiraceae showed comparable relative abundances in BP-only and mixed-infection groups. In contrast, mixed infections were characterized by a relative increase in *Vibrio*, together with the PCR-detection of PirAB toxin genes, whereas WSSV remained detectable in the metagenomic dataset despite its comparatively low relative abundance, while the infection remained thermally suppressed and asymptomatic in the larvae.
214-215	**In the Results section**<br>Additionally, *Vibrio* was 3.2-fold more abundant in the mixed-infection group than in the other health status.	261-266	**In the Results section**<br>Overall, *Vibrio* represented a relatively small fraction of the shrimp microbiome (1.3% to 0.18%), although its relative abundance and community composition varied among health statuses. A relative increase in *Vibrio* was observed in the mixed-infection group, reaching a 3.2-fold higher abundance than in the non-infected group. In contrast, *Vibrio* abundance was lower in the BP-only group than in the non-infected group. Despite this lower abundance, *Vibrio* community composition differed significantly between BP-only and non-infected groups (PERMANOVA, $p = 0.045$).
263-268	**In the Discussion section**<br>The consistent detection of BP in mixed infection with bacterial pathogens has been reported previously, suggesting a possible synergistic interaction in which bacterial toxins or local inflammatory processes may favor viral replication or the formation of inclusion bodies [19]. In our study, we observed two infection patterns across tanks: some tanks contained BP-infected larvae, while others showed mixed infections, with larvae harboring BP and others affected by WSSV and AHPND. Histology and PCR confirmed mixed infections, which were associated with an enriched *Vibrio* microbiome compared with the non-infected and BP-infected groups. High mortality was restricted to mixed-infection tanks, indicating that BP alone does not drive mortality under these conditions. Instead, BP may predispose larvae to secondary pathogens. Since WSSV and AHPND-causing bacteria were never detected together without the presence of BP, controlled challenge experiments are required to validate this hypothesis.	325-342	**In the Discussion section**<br>The consistent detection of BP in mixed infection with bacterial pathogens has been reported previously, suggesting a possible synergistic interaction in which bacterial toxins or local inflammatory processes may favor viral replication or the formation of inclusion bodies [19]. In our study, we observed two infection patterns across tanks: BP infection alone and mixed-infection conditions in which BP occurred together with WSSV and/or AHPND-causing bacteria. These infection patterns were supported by histological, molecular, and metagenomic evidence at the tank level. Mixed-infection tanks showed a relative increase in *Vibrio* compared with both BP-infected and non-infected groups. High mortality was restricted to mixed-infection tanks, indicating that BP alone was not linked to high mortality under these conditions. Although BP infection alone was associated with the lowest *Vibrio* abundance, *Vibrio* community composition differed significantly between BP-infected and non-infected groups, suggesting that BP may be associated with changes in *Vibrio* community structure rather than with increased overall abundance. Because WSSV and AHPND-causing bacteria were not detected together in the absence of BP in this observational dataset, whether BP facilitates subsequent pathogen establishment or simply co-occurs under shared predisposing conditions cannot be determined. Controlled challenge experiments will therefore be required to test these potential interactions.

Reviewer 1 point #14: In this study, AHPND-causing bacteria were identified via the AP4 primer (targeting the PirAB toxin) and metagenomic PirAB gene abundance. Was the Vibrio species itself confirmed (e.g., via ANI/marker gene) as V. parahaemolyticus as well? Since AHPND can also be caused by other Vibrio species carrying the same toxin plasmid?

Author response #14: We thank the reviewer for this important observation. This issue has been addressed in detail in our response to Editor point #6.

Reviewer 1 point #15:

Minor comments
- "...showing a 3.3-fold higher abundance than in the non-infected groups." – should be singular, "non-infected group," since only one non-infected group is defined in this study.
- Please check. The in-text citation numbers do not consistently match the numbered reference list from roughly reference [35] onward. For example, the BP primer set is cited as [35], but reference 35 is the EHP nested-PCR paper (Jaroenlak et al.) – the actual BP-PCR paper (Wang et al.) is reference 34. Similarly, SqueezeMeta is cited as [40] but reference 40 in the list is MEGAHIT (Li et al.), and CheckM is cited as [47] but reference 47 is VAPiD (Shean et al.). This off-by-one pattern appears to run through most of the Materials and Methods and later sections and should be carefully checked and corrected throughout the manuscript.
- Please define the post-larval stage abbreviations (PL9, PL10, PL11) at first use, as readers outside the shrimp aquaculture field may not be familiar with this nomenclature.

Author response #15: We thank the reviewer for these observations. All of these minor comments have been addressed and corrected throughout the revised manuscript.

Reviewer 2

Reviewer 2 point #1: Line 17: Please add the WSSV and AHPND meaning.
Author response #1: The meaning has been added to the first time in the abstract section

Reviewer 2 point #2: Line 45: please correct to were significantly lower during retting
Author response #2: The sentence has been revised accordingly.

Reviewer 2 point #3: Line 40-41: Drafting of the first highlight should be modified: “Reduced and distinct microbiome structures were observed in shrimp infected with BP and in those with mixed infections” may be improved, please add which microbiome structures.
Author response #3: We thank the reviewer for this observation. The highlight has been revised to specify that BP and mixed infections were associated with reduced microbial richness and diversity and distinct community structures.

Reviewer 2 point #4: Figure captions: please double check the correct spelling and the name of species should be in italics for example: Baculovirus penaei
Author response #4: We thank the reviewer for this observation. All figure captions were carefully reviewed for correct spelling and species nomenclature, and scientific names, including *Baculovirus penaei*, have been italicized where appropriate.

Reviewer 2 point #5: Conclusions: Please add a short paragraph regarding your last finding respect the findings of the assembly of the complete BP genome in your study, and that the in-silico alignment provided a molecular explanation for the discrepancy found, reenforcing the critical need to refine molecular tools for future larval microbiome research to ensure accurate pathogen correlation.
Author response #5: We thank the reviewer for this valuable suggestion. We have added a short paragraph to the Discussion highlighting the significance of the near-complete BP-GR25 genome assembled in this study. We also emphasize how the in silico primer-alignment analysis provided a molecular explanation for the observed diagnostic discrepancy and underscores the need to refine and validate molecular detection tools to improve pathogen identification and correlation in future larval microbiome studies.

Reviewer 2 point #6: References: Please correct the species’ names; they should be in italics, and the year of publication in bold.
Author response #6: The references have been corrected.
