# Supplementary material for: Microbial community structure of Penaeus vannamei post-larvae naturally infected with multiple viruses in hatchery tanks
Source: PLoS One. 2026 Oct 27;21(10):e0361576. doi: 10.1371/journal.pone.0361576 (PMC13712688; doi:10.1371/journal.pone.0361576)
Supplement: Peer review history

# Peer review: Decision Letter 0

**DOI:** 10.1371/journal.pone.0361576.r001

**Authors:** Adrian Ochoa-Leyva

20 Aug 2026

Dear Dr. Reyes Román,

The two reviewers recognize the potential value of this study, particularly the integration of histopathology, pathogen diagnostics, survival data, shotgun metagenomics, and the recovery of a *Baculovirus penaei*  genome from commercial hatchery samples. However, Reviewer 1 has raised several important concerns regarding the experimental design, statistical analyses, metagenomic methodology, and interpretation of the results. After carefully evaluating the manuscript and the reviewers’ comments, I agree that substantial revision is required before the manuscript can be considered further.

Please submit your revised manuscript within Oct 19 2026 11:59PM. If you will need more time than this to complete your revisions, please reply to this message or contact the journal office at plosone@plos.org. When you're ready to submit your revision, log on to https://www.editorialmanager.com/pone/ and select the 'Submissions Needing Revision' folder to locate your manuscript file.

Please include the following items when submitting your revised manuscript:

- A letter that responds to each point raised by the academic editor and reviewer(s). You should upload this letter as a separate file labeled 'Response to Reviewers'.
- A marked-up copy of your manuscript that highlights changes made to the original version. You should upload this as a separate file labeled 'Revised Manuscript with Track Changes'.
- An unmarked version of your revised paper without tracked changes. You should upload this as a separate file labeled 'Manuscript'.

If you would like to make changes to your financial disclosure, please include your updated statement in your cover letter. Guidelines for resubmitting your figure files are available below the reviewer comments at the end of this letter.

If applicable, we recommend that you deposit your laboratory protocols in protocols.io to enhance the reproducibility of your results. Protocols.io assigns your protocol its own identifier (DOI) so that it can be cited independently in the future. For instructions see: https://journals.plos.org/plosone/s/submission-guidelines#loc-laboratory-protocols. Additionally, PLOS One offers an option for publishing peer-reviewed Lab Protocol articles, which describe protocols hosted on protocols.io. Read more information on sharing protocols at https://plos.org/protocols?utm_medium=editorial-email&utm_source=authorletters&utm_campaign=protocols.

As the corresponding author, your ORCID iD is verified in the submission system and will appear in the published article. PLOS supports the use of ORCID, and we encourage all coauthors to register for an ORCID iD and use it as well. Please encourage your coauthors to verify their ORCID iD within the submission system before final acceptance, as unverified ORCID iDs will not appear in the published article. *Only*  the individual author can complete the verification step; PLOS staff *cannot*  verify ORCID iDs on behalf of authors.

We look forward to receiving your revised manuscript.

Kind regards,

Adrian Ochoa-Leyva

Academic Editor

PLOS One

Journal Requirements:

When submitting your revision, we need you to address these additional requirements.

1. Please ensure that your manuscript meets PLOS ONE's style requirements, including those for file naming. The PLOS ONE style templates can be found at https://journals.plos.org/plosone/s/file?id=wjVg/PLOSOne_formatting_sample_main_body.pdf and https://journals.plos.org/plosone/s/file?id=ba62/PLOSOne_formatting_sample_title_authors_affiliations.pdf

2. Please include a separate caption for each figure in your manuscript, and remove the separate file.

3. Please include captions for your Supporting Information files at the end of your manuscript, and update any in-text citations to match accordingly. Please see our Supporting Information guidelines for more information: http://journals.plos.org/plosone/s/supporting-information.

If the reviewer comments include a recommendation to cite specific previously published works, please review and evaluate these publications to determine whether they are relevant and should be cited. There is no requirement to cite these works unless the editor has indicated otherwise.

Additional Editor Comments:

In addition to addressing **all comments raised by both reviewers** , please pay particular attention to the following editorial points:

1. **Observational design and causal interpretation**
  This study is based on an observational comparison of commercial production tanks with an unbalanced number of replicates among groups. Therefore, the current data support associations between infection status, microbiome composition, and survival, but do not establish causality or pathogen synergism. Statements indicating that mixed infection “drives” mortality, that BP predisposes larvae to secondary infection, that synergistic pathogenicity was demonstrated, or that BP replication is enhanced should be substantially revised throughout the Abstract, Results, Discussion, Highlights, and Conclusions. These observations may be presented as hypotheses that require validation through controlled infection experiments.
2. **Experimental unit, tank-level metadata, and possible confounding variables**
  Please explicitly state that the **tank is the experimental/statistical unit** , whereas the post-larvae analyzed from each tank represent a pooled biological sample. The approximate number of post-larvae contributing to each ~1 g sample should also be reported.
  In addition, please provide a comprehensive supplementary table containing, for each of the 18 tanks, survival, infection status, sampling information, and all available environmental/production variables, including temperature, salinity, pH, dissolved oxygen, stocking density, water source, and any relevant management practices. These variables should be statistically compared among groups whenever the data are available.
3. **Host DNA removal and effective microbial sequencing depth**
  The manuscript states that host-derived contigs were removed, but the host-depletion strategy is insufficiently described. Please provide full methodological details, including the reference genome used, filtering criteria, whether filtering was performed at the read or contig level, and the software/parameters employed. It is necessary to report the number of final reads used for the assembly and the calculation of the abundances compared to the initial reads, and the number of reads that mapped to shirmp and human genomes.
  Importantly, provide per-sample statistics including raw reads, reads retained after quality control, host-associated reads, microbial reads, viral reads where applicable, mapped reads, and mapping percentages.
4. **Validation of the BP-GR25 genome and the claim of genome completeness**
  The manuscript refers to BP-GR25 as a “complete genome”; however, the evidence currently presented is insufficient to establish completeness. In particular, CheckM is primarily designed for bacterial and archaeal genome quality assessment and cannot by itself validate the completeness of a nudivirus genome. Please provide virus-appropriate evidence supporting genome completeness, including the number of contigs comprising BP-GR25, coverage across the genome, assembly-graph or junction evidence where applicable, and the presence of expected conserved nudiviral core genes.
  If genome closure or completeness cannot be conclusively demonstrated, please use a more conservative designation such as **“near-complete genome”**  or **“metagenome-assembled consensus genome.”**
5. **Per-sample validation and strain variation of BP-GR25**
  Because BP-GR25 was recovered from a co-assembly, please determine whether the assembled sequence represents a single strain or a consensus derived from genetically distinct BP populations across tanks. Reads from each BP-positive and mixed-infection sample should be independently mapped against BP-GR25. Please provide, for each sample, breadth and depth of coverage and, where possible, an assessment of sequence variation/SNP density. This analysis is also important to exclude possible assembly artifacts or chimeric regions and to evaluate strain-level variation among tanks.
6. **Identification of AHPND-associated bacteria and interpretation of**  ***Vibrio***
  Detection of the PirAB-associated AP4 target establishes the presence of AHPND-associated genetic determinants but does not necessarily establish *Vibrio parahaemolyticus*  as the carrier, since AHPND can be associated with different *Vibrio*  species. If the metagenomic data permit, please determine the taxonomic identity of the PirAB-carrying organism using appropriate genomic or marker-based evidence. Otherwise, the manuscript should consistently use terminology such as **“AHPND-causing bacteria”**  and avoid attributing the phenotype specifically to *V. parahaemolyticus*  without direct evidence.

Finally, please carefully review the manuscript for internal inconsistencies noted by the reviewers, including sequencing-platform information, total and mean read counts, reference numbering, diagnostic terminology, and the discrepancy between statements indicating that no additional microorganisms were detected and subsequent reporting of additional bacterial taxa.

I would like to emphasize that I do **not**  consider new controlled infection experiments essential for reconsideration of the present manuscript, provided that the authors appropriately restrict their conclusions to what can be supported by this observational dataset. However, the statistical, bioinformatic, genomic, and interpretative issues outlined above must be satisfactorily addressed.

[Note: HTML markup is below. Please do not edit.]

Reviewers' comments:

Reviewer's Responses to Questions

**Comments to the Author**

1. Is the manuscript technically sound, and do the data support the conclusions?

Reviewer #1: Yes

Reviewer #2: Yes

**********

2. Has the statistical analysis been performed appropriately and rigorously?

Reviewer #1: No

Reviewer #2: Yes

**********

3. Have the authors made all data underlying the findings in their manuscript fully available?

The PLOS Data policy

Reviewer #1: Yes

Reviewer #2: Yes

**********

4. Is the manuscript presented in an intelligible fashion and written in standard English?

Reviewer #1: Yes

Reviewer #2: Yes

**********

**Reviewer #1:**  Manuscript ID: PONE-D-26-29303

Manuscript title: Microbial community structure of Penaeus vannamei post-larvae naturally infected with multiple viruses in hatchery tanks

Overview

This manuscript reported a potentially useful field-based metagenomic dataset from 18 commercial shrimp-hatchery tanks and also presented an assembled Baculovirus penaei genome. The effort to integrate histopathology, PCR diagnostics, survival, and shotgun metagenomics is a strength of this study. However, the current manuscript over-interprets an observational, unbalanced tank-level comparison as evidence of pathogen-specific and synergistic causal effects. Important details of the sampling design, metagenomic workflow, statistical analysis, and genome validation are also missing. I suggest authors to add the important details and revise to improve quality and clarity of this study.

Comments

1. Please add geographic location of the sampling sites, this will help readers to see distribution of the 18 field sites.

2. The study compares 11 BP-positive tanks, four mixed-infection tanks, and three non-infected tanks, which is quite interesting. However, the proportion in the mixed group ,containing BP, WSSV and AHPND, could not be quantitatively measured, along with potential tank-effect as an additional factor. Therefore, statements that mixed infection “drives” mortality, BP predisposes larvae to secondary infection, or synergistic pathogenicity was demonstrated should be revised perhaps, softened and presented as hypotheses for a future controlled challenge trial.

3. Please add % survival of each individual tank from each group (Table 1), and define whether reported variation was SD or SEM values (Table 1). The authors should also perform statistical comparison of %survival. It’s also very important to provide water parameters of each tank and also stat tests of each key parameter to explore whether environmental factors play roles in different survival levels or not.

4. Please verify the total read yield and per-sample average: the manuscript states 1,498 million clean reads across the 18 samples, “averaging 74 million reads per sample”, but 1,498 / 18 ≈ 83 million. Please correct whichever figure is inaccurate.

5. The sequencing platform is reported inconsistently: the Abstract states microbiome profiling was done “on the Illumina NovaSeq X plus platform,” while the Methods section (“DNA extraction and microbiome sequencing”) states sequencing “was performed on an Illumina NovaSeq 6000 platform.” Please confirm which instrument was actually used and correct throughout.

6. In Fig. 4, infected samples show COG enrichment for retrotransposon proteins, transposases, and reverse transcriptases, please discuss whether these genes encoded by baculoviruses or nudiviruses or microbes. Or do they reflect residual host (shrimp) genomic DNA contamination in the metagenome? Please clarify this point explicitly, as distinguishing between host contamination and bacterial dysbiosis substantially alters the biological interpretation.

7. Please clarify the unit of replication used for microbiome sequencing: how many post-larvae were pooled into the ~1 g subsample per tank that was preserved for HTS? This matters for interpreting whether the diversity/statistical comparisons treat tanks or pooled individuals as the sampling unit.

8. Gene abundances were normalized as “Transcripts Per Million (TPM)”, but this is a shotgun DNA metagenomic dataset rather than an RNA-seq/transcriptomic one. Please clarify or correct this terminology (e.g., reads-per-million or a similarly DNA-appropriate metric) if “transcripts” was carried over from RNA-seq convention in error.

9. In Table 1, what the “2 Positive / 9 Negative (BP)” and “BP (1/4)” notations represent (i.e., PCR-positive tanks out of total tanks tested per group), since the column header lists three pathogens (WSSV/AHPND/BP) but the BP-positive row only reports one ratio.

10. The Discussion and Conclusion both describe WSSV as showing "high genomic abundance" despite covert/asymptomatic infection, but Fig 3B shows WSSV at a much lower relative abundance — far below Baculovirus. The authors should explain what "high" means here (e.g., high relative to non-infected, or high viral copy number despite low relative abundance in the whole metagenome) so this isn't read as contradictory.

11. In the result section, 11 tanks were histologically positive for BP (tetrahedral occlusion bodies), but PCR confirmed only 2/11 (Table 1). The authors mentioned that this could be related to primer mismatch (BPB–BPG vs. the sequenced strain). However, could it also be due to the possibility that some "tetrahedral occlusion bodies" seen histologically could be a non-BP artifact, given the low PCR confirmation rate as well. Please elaborate and discuss this.

12. The BP-GR25 genome was recovered via co-assembly/binning across pooled metagenomes from multiple tanks (both BP-only and mixed-infection groups). Was strain-level variation among tanks assessed, or does BP-GR25 represent a single consensus that could be masking real strain diversity across the 15 infected tanks?

13. The manuscript reports Vibrio as "3.2-fold more abundant in mixed infection than in other health status," can the authors explain why Vibrio was also increased in BP-only tanks relative to non-infected ones. Given the hypothesis that BP predisposes larvae to secondary bacterial infection, this specific comparison (BP-only vs. non-infected) is also important to report and discuss.

14. In this study, AHPND-causing bacteria were identified via the AP4 primer (targeting the PirAB toxin) and metagenomic PirAB gene abundance. Was the Vibrio species itself confirmed (e.g., via ANI/marker gene) as V. parahaemolyticus as well? Since AHPND can also be caused by other Vibrio species carrying the same toxin plasmid?

Minor comments

- “...showing a 3.3-fold higher abundance than in the non-infected groups.” – should be singular, “non-infected group,” since only one non-infected group is defined in this study.

- Please check. The in-text citation numbers do not consistently match the numbered reference list from roughly reference [35] onward. For example, the BP primer set is cited as [35], but reference 35 is the EHP nested-PCR paper (Jaroenlak et al.) – the actual BP-PCR paper (Wang et al.) is reference 34. Similarly, SqueezeMeta is cited as [40] but reference 40 in the list is MEGAHIT (Li et al.), and CheckM is cited as [47] but reference 47 is VAPiD (Shean et al.). This off-by-one pattern appears to run through most of the Materials and Methods and later sections and should be carefully checked and corrected throughout the manuscript.

- Please define the post-larval stage abbreviations (PL9, PL10, PL11) at first use, as readers outside the shrimp aquaculture field may not be familiar with this nomenclature.

**Reviewer #2:**  The manuscript presented by Andrade et al., titled “Microbial community structure of Penaeus vannamei post-larvae naturally infected with multiple viruses in hatchery tanks.” presents very interesting molecular results related to assess commercial larval production tanks infected by polymicrobial pathogenesis. The authors used

Adequate molecular and histological tools that supports their results of the shrimp’s infections provoked by: Baculovirus penaei (BP) monoinfection, mixed infections (BP, WSSV, and AHPND causing bacteria), as well their results were compared with a control group. The principal results were assessed profiling the larval

microbiome via shotgun metagenomic sequencing on the Illumina NovaSeq X plus platform in order to broader the ecological impacts which are very important in economic and ecological aquaculture shrimp performance, moreover, the importance of present a polymicrobial pathogenesis study focused in shrimp microbiota has a very significant findings that will be of interest for shrimp microbiology studies, for these reasons, I consider that this paper could be accepted for publication in this journal with minor revisions.

Here are some corrections and recommendations to take in account to be published:

Line 17: Please add the WSSV and AHPND meaning.

Line 45: please correct to were significantly lower during retting

Line 40-41: Drafting of the first highlight should be modified: “Reduced and distinct microbiome structures were observed in shrimp infected with BP and in those with mixed infections” may be improved, please add which microbiome structures.

Figure captions: please double check the correct spelling and the name of species should be in italics for example: Baculovirus penaei

Conclusions: Please add a short paragraph regarding your last finding respect the findings of the assembly of the complete BP genome in your study, and that the in-silico alignment provided a molecular explanation for the discrepancy found, reenforcing the critical need to refine molecular tools for future larval microbiome research to ensure accurate pathogen correlation.

References: Please correct the species’ names; they should be in italics, and the year of publication in bold.

**********

what does this mean?). If published, this will include your full peer review and any attached files.

If you choose “no”, your identity will remain anonymous but your review may still be made public.

**Do you want your identity to be public for this peer review?**  For information about this choice, including consent withdrawal, please see our Privacy Policy

Reviewer #1: No

Reviewer #2: **Yes:**  Kadiya Calderon

**********

[NOTE: If reviewer comments were submitted as an attachment file, they will be attached to this email and accessible via the submission site. Please log into your account, locate the manuscript record, and check for the action link "View Attachments". If this link does not appear, there are no attachment files.]

To ensure your figures meet our technical requirements, please review our figure guidelines: https://journals.plos.org/plosone/s/figures

You may also use PLOS’s free figure tool, NAAS, to help you prepare publication quality figures: https://journals.plos.org/plosone/s/figures#loc-tools-for-figure-preparation.

NAAS will assess whether your figures meet our technical requirements by comparing each figure against our figure specifications.

# Author comment: Author response to Decision Letter 1

**DOI:** 10.1371/journal.pone.0361576.r002

15 Sep 2026

Due to the platform’s limitations, it was not possible to include the complete information in the online response fields. We therefore kindly ask the Editor and Reviewers to consult the attached "Response to Reviewers" document, where all comments have been addressed in detail and the corresponding revisions are fully described.

Attachment Submitted filename: Response to Reviewers.pdf

# Peer review: Decision Letter 1

**DOI:** 10.1371/journal.pone.0361576.r003

**Authors:** Adrian Ochoa-Leyva

14 Oct 2026

Microbial community structure of Penaeus vannamei post-larvae naturally infected with multiple viruses in hatchery tanks.

PONE-D-26-29303R1

Dear Dr. Reyes Román,

We’re pleased to inform you that your manuscript has been judged scientifically suitable for publication and will be formally accepted for publication once it meets all outstanding technical requirements.

Within one week, you’ll receive an e-mail detailing the required amendments. When these have been addressed, you’ll receive a formal acceptance letter and your manuscript will be scheduled for publication.

An invoice will be generated when your article is formally accepted. Please note, if your institution has a publishing partnership with PLOS and your article meets the relevant criteria, all or part of your publication costs will be covered. Please make sure your user information is up-to-date by logging into Editorial Manager at Editorial Manager® and clicking the ‘Update My Information' link at the top of the page. For questions related to billing, please contact billing support.

If your institution or institutions have a press office, please notify them about your upcoming paper to help maximize its impact. If they’ll be preparing press materials, please inform our press team as soon as possible -- no later than 48 hours after receiving the formal acceptance. Your manuscript will remain under strict press embargo until 2 pm Eastern Time on the date of publication. For more information, please contact onepress@plos.org.

Kind regards,

Adrian Ochoa-Leyva

Academic Editor

PLOS One

Additional Editor Comments (optional):

Reviewers' comments:

Reviewer's Responses to Questions

**Comments to the Author**

Reviewer #2: All comments have been addressed

Reviewer #3: All comments have been addressed

**********

2. Is the manuscript technically sound, and do the data support the conclusions?

Reviewer #2: Yes

Reviewer #3: Yes

**********

3. Has the statistical analysis been performed appropriately and rigorously?

Reviewer #2: Yes

Reviewer #3: Yes

**********

4. Have the authors made all data underlying the findings in their manuscript fully available?

The PLOS Data policy

Reviewer #2: Yes

Reviewer #3: Yes

**********

5. Is the manuscript presented in an intelligible fashion and written in standard English?

Reviewer #2: Yes

Reviewer #3: Yes

**********

Reviewer #2: Authors  have positively addressed the most of the queries raised during the first round of review. However, some more attention is to be given concerning formatting of manuscript as per the journal style before the final publications

Reviewer #3: The authors have successfully addressed all the reviewers’ comments. The revisions have improved the clarity of the manuscript, strengthened the presentation and interpretation of the results, and provided more robust support for the conclusions.

**********

what does this mean?). If published, this will include your full peer review and any attached files.

If you choose “no”, your identity will remain anonymous but your review may still be made public.

**Do you want your identity to be public for this peer review?**  For information about this choice, including consent withdrawal, please see our Privacy Policy

Reviewer #2: **Yes:**  Kadiya Calderon

Reviewer #3: No

**********

# Editor report: Acceptance letter

**DOI:** 10.1371/journal.pone.0361576.r004

**Authors:** Adrian Ochoa-Leyva

PONE-D-26-29303R1

PLOS One

Dear Dr. Reyes,

I'm pleased to inform you that your manuscript has been deemed suitable for publication in PLOS One. Congratulations! Your manuscript is now being handed over to our production team.

At this stage, our production department will prepare your paper for publication. This includes ensuring the following:

* All references, tables, and figures are properly cited

* All relevant supporting information is included in the manuscript submission,

* There are no issues that prevent the paper from being properly typeset

You will receive further instructions from the production team, including instructions on how to review your proof when it is ready. Please keep in mind that we are working through a large volume of accepted articles, so please give us a few days to review your paper and let you know the next and final steps.

Lastly, if your institution or institutions have a press office, please let them know about your upcoming paper now to help maximize its impact. If they'll be preparing press materials, please inform our press team within the next 48 hours. Your manuscript will remain under strict press embargo until 2 pm Eastern Time on the date of publication. For more information, please contact onepress@plos.org.

You will receive an invoice from PLOS for your publication fee after your manuscript has reached the completed accept phase. If you receive an email requesting payment before acceptance or for any other service, this may be a phishing scheme. Learn how to identify phishing emails and protect your accounts at https://explore.plos.org/phishing.

If we can help with anything else, please email us at customercare@plos.org.

Thank you for submitting your work to PLOS One and supporting open access.

Kind regards,

PLOS One Editorial Office Staff

on behalf of

Dr. Adrian Ochoa-Leyva

Academic Editor

PLOS One
